# Change in Formal and Informal Forest Management Institutions Induced by Health Shocks—A Global Systematic Review

**DOI:** 10.1007/s00267-025-02250-x

**Published:** 2025-09-03

**Authors:** Ametus Kuuwill, Jude Ndzifon Kimengsi, Lukas Giessen

**Affiliations:** 1 Forest Institutions and International Development (FIID) Research Group, Chair of Tropical and International Forestry, Faculty of Environmental Sciences, Technische Universität Dresden, Tharandt, Germany; 2 Chair of Tropical and International Forestry, Faculty of Environmental Sciences, Technische Universität Dresden, Tharandt, Germany

**Keywords:** Formal institutions, Informal institutions, Ebola, COVID-19, Institutional change, Outcomes

## Abstract

Studies on the impact of health shocks in (re)shaping forest management institutions exist, albeit fragmented. Similarly, significant knowledge gaps exist regarding conceptualizing health shocks, the mechanisms and outcomes of forest-linked institutional change, and the methods used so far. We review regional variations in conceptualizing forest management institutions and institutional change that are linked to health shocks. Further, we studied the mechanism of institutional change and outcome in the context of health shocks and evaluated the yet-to-be-filled methodological gaps. Using the critical eco-health approach and an institutional analysis framework, we systematically review 70 empirically conducted studies. Descriptive and directed content analysis was employed in the data analysis. First, we found that health shocks are predominantly conceptualized as pandemics in Asia and epidemic in Africa. Forest management institutions are viewed through the process dimension lens, with informal processes more prevalent in Africa and formal processes dominant in other regions. Second, health shocks have primarily induced new formal forest management institutions while eroding informal ones in Asia and Africa. Thirdly, these institutional changes are linked with negative ecological and economic outcomes in the developing subregions, particularly in Asia, followed by Africa and Latin America. Finally, most studies employed the qualitative and single case study approaches, potentially limiting the findings’ generalizability. Our study establishes a gap in understanding the power dynamics and political process of institutional change in the context of health shocks. Future studies should use a multiple-case study approach, mixed methods, and actor-centred analysis of forest management institutional compliance during health shocks.

## Introduction

The global landscape is marked by a staggering net forest loss of 178 million hectares, about the size of Libya (FAO 2020). For the tropics, forest landscape transformation stands at 12 million hectares (Garcia et al. 2020; Kimengsi et al. 2023). Anthropogenic activities are primarily identified as the primary drivers of this transformation (Houghton and Nassikas 2018; Li et al. 2021). For instance, studies underscore the role of forests in sustaining the livelihoods of about 1.6 billion people worldwide; some 350 million people, mainly in the global south, depend on the forest substantially (Chao 2012; Newton et al. 2020). Hence, rapid forest landscape transformation concerns are usually linked to livelihood vulnerabilities, conflicts, and migration (Anthonia et al. 2021; Chirwa and Adeyemi 2020). While these debates occupy the literature and policy domain, forest use and human health issues, synonymously referred to as health shocks hereafter, due to the sudden health, economic, and livelihood impact on households (Sinclair and Smetters 2004), are under-explored.

Some scholars argue that forest houses pathogens and other disease hosts, transferring several infectious diseases such as Malaria, Onchocerciasis, and Brugian filariasis (Chang et al. 1991; Lainson 1983; Marrelli et al. 2007). These arguments deductively suggest forest clearance, staying at a distance from forested areas, or migration from forested communities as disease mitigation strategies. To this end, Rahman et al. (2020) found that about 60% of all infectious diseases in humans and 75% of all emerging contagious diseases originate from forest ecosystems through human-wildlife contact. For example, HIV, the virus responsible for AIDS, recognized in 1983, was found to have been transmitted through human interaction with chimpanzees (Devaux et al. 2019; Keele et al. 2006). Human-wildlife interaction was identified as the primary conduit for the spread of Severe Acute Respiratory Syndrome (SARS) in China in 2002, with the coronavirus (CoV) as its causative agent (Gully 2020). The emergence of H1N5 influenza in Hong Kong in 1996, which resurged in 2003, and the H1N1 pandemic in 2009 in Mexico were found to be of zoonotic origins (Gully 2020). The Ebola virus outbreak in Africa in 1972, which claimed the lives of about 11,308 Africans between 2014 and 2017, was believed to have originated from human-primates contact (Barbiero 2020). Furthermore, the fairly recent coronavirus 2 (SARS-CoV-2), known as the COVID-19 pandemic, which started in Wuhan, China, is asserted to have emerged from human-wildlife contact, although not fully substantiated (Tiwari et al. 2020; WHO 2024). However, the dilution hypothesis suggests that forest biodiversity helps mitigate diverse adverse health conditions and should be conserved (Hayman et al. 2013; Karesh et al. 2012). Several studies have noted the medicinal role of forests, their psychological therapeutic functions, and nutritional contributions, which help human immune systems in fighting adverse health conditions (Bai et al. 2019; De Meyer et al. 2022).

While the debate on forest and health remains scanty and fragmented, evidence indicates high unemployment, disruption of the international supply chain, and economic downturns, among others, as the devastating brunt of these public health crises at the macro level (Aduhene and Osei-Assibey 2021; ILO 2020). At the micro level, health shocks have (re)shaped resource-dependent communities (Pretzsch et al. 2014; Shackleton et al. 2011). With these arguments, the literature on health shocks and forest use and management is skewed toward profiling rural livelihood vulnerabilities and forest dependencies (Laudari et al. 2021). For instance, Kuuwill et al. (2022) suggest that communities rely on forest resources as natural insurance or capital to offset economic burdens during health shocks. Studies in Asia and the Brazilian Amazon show high dependence on forests for medicine, food, and income during times of health shocks (Atin and Lintangah 2023; Rahimian et al. 2022; Vale et al. 2021). Also, empirical evidence in Africa highlights that households either diversify or intensify their livelihood activities when faced with health shocks (Kuuwill et al. 2022; Mbiba et al. 2018; Saxena et al. 2021).

Nevertheless, forest-linked activities are conducted within a society’s governance architecture, emphasizing the roles of formal and informal forest management institutional framework (Giessen and Buttoud 2014; Kimengsi et al. 2020). Defined as the rules of the game, formal or informal institutions have mediated resource access, use, and management by acting as constraints or enabling social fabrics (Fleetwood 2008; Ostrom 2009). While scholars agree on the importance of institutions in resource management, their conceptualizations vary, underscoring the need for scientific focus on how institutions are conceptualized (Kimengsi and Mukong 2023). This is particularly important in the context of health shocks, which (un)consciously (re)shape forest use behaviors (Kuuwill and Kimengsi 2023; Kuuwill et al. 2025). For instance, institutions are viewed as processes: rules, conventions, laws, norms, and traditions that regulate social relations (Hodgson 2006; Lomazzi 2023). Yet, others point to the visible social arrangements, such as departments, ministries, and local forest management groups, as institutions (Nysten-Haarala 2013; Peters 2014).

Amidst these conceptual divergences, what is certain is that institutions do change (Brousseau et al. 2011; Greif and Laitin 2004; Mahoney and Thelen 2010). Such changes are argued to be either precipitated by endogenous events that gradually undermine institutional fabrics or, in other scenarios, abrupt exogenous events that propel rapid reactive institutional change (Gerschewski 2021a; Mahoney and Thelen, 2010). This implies that a marginal change in the status quo can trigger a shift in institutions mediating resource access, use, and management with varying ecological, economic, political, and social outcomes (Kimengsi et al. 2024; Owusu et al. 2024). For example, studies found that changes in populations and youth preference of resource-dependent communities significantly led to forest management institutional change in Ethiopia and Cameroon over time (Kimengsi, Mukong, et al. 2023; Wakjira et al. 2013). Others highlight the sudden price increase of forest products due to abrupt global market demands that trigger a rapid institutional change response (van Kooten and Schmitz 2022). Yet, some scholarships indicate that forest-linked institutional change is triggered by environmental challenges, resource scarcity, and conflict over resources, which may be sudden or over time (Munck af Rosenschöld et al. 2014; Turner 1999).

While these debates exist, the mechanisms of institutional change have either been relegated or under-explored. Further, amidst these unsettling debates, a comprehensive understanding of how health shocks shape forest-linked formal and informal institutions guiding resource use behaviors towards sustainable pathways remains an aperture in the literature (Acquaah et al. 2024; Kuuwill et al. 2024). Against this backdrop, this paper systematically reviews the literature on health shocks and forest-linked institutions to address the following questions: (i) How are health shocks and forest-linked institutions conceptualized in the literature? (ii) What is the state of knowledge on the mechanisms of forest-linked institutional change induced by health shocks? (iii) What are the management outcomes of forest-linked institutions shaped by health shocks? and (iv) What methodologies have been employed in analyzing health shocks and forest-linked institutions? Insights from this study are crucial for informing future research and policy endeavors aimed at rethinking and developing health-resilient institutions toward forest sustainability and resilient livelihood systems (Acquaah et al. 2023; Tamakloe et al. 2025).

## Materials and Methods

### Analytical Framework

**Fig. 1:**
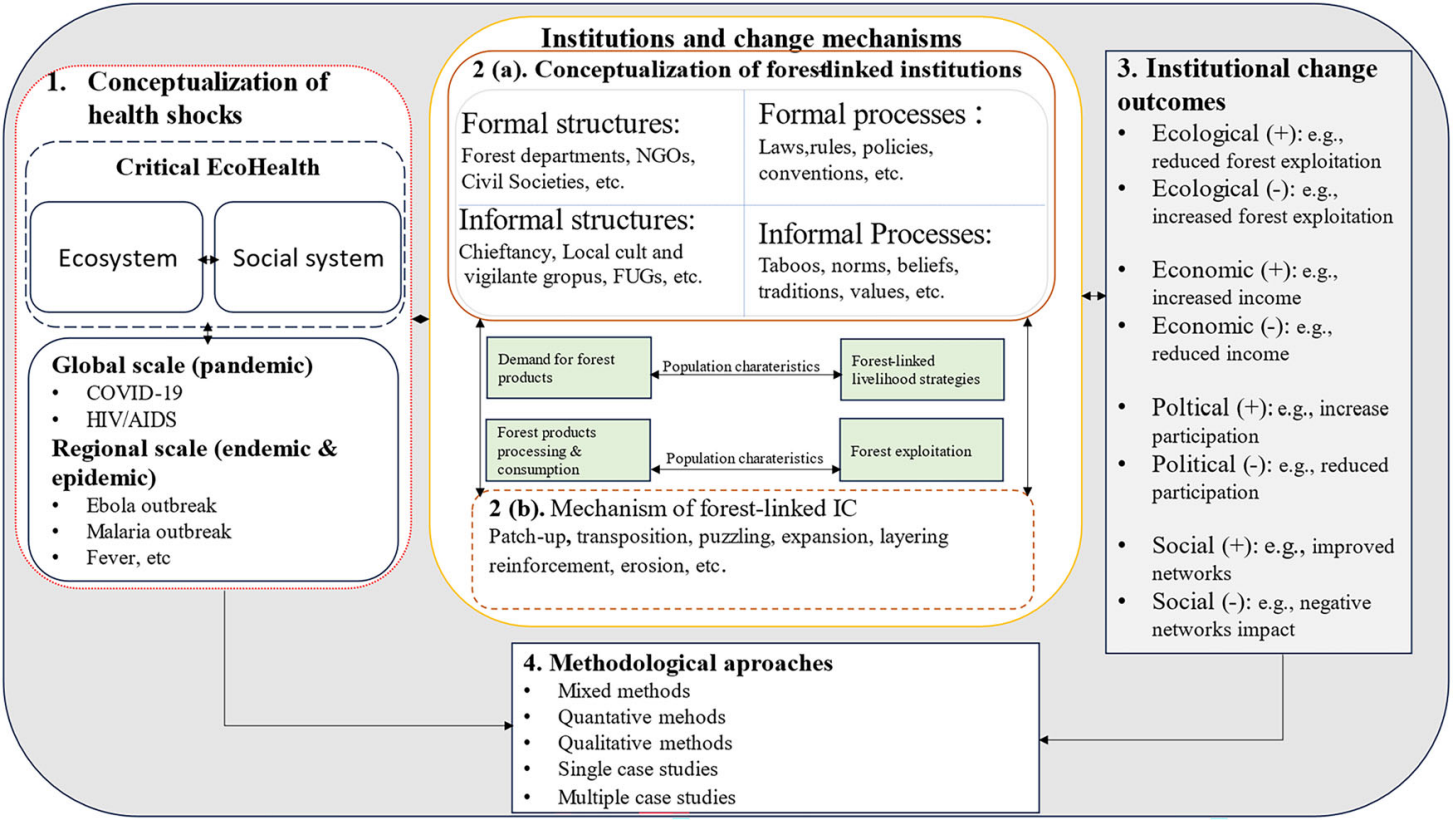
Analytical framework for analyzing health shocks, forest-linked institutional change, and forest management outcomes, developed based on Koning (2016), Kimengsi et al. (2022), Krott et al. (2014), and Lebel (2003)

The study draws its analytical framework from the critical eco-health approach and the analytical lens on institutions (Fig. 1). The traditional eco-health approach emerged on the premise that humans eke out livelihoods from the ecosystem (Harrison et al. 2019). Doing so increases interaction between human social and ecological systems, noted to house disease pathogens, leading to disease outbreaks affecting human health and well-being (Hayman et al. 2013; Lebel 2003). While some of these diseases spread at regional scales (e.g., endemics^1^ and epidemics^2^), others spread globally (pandemics^3^). Using the critical eco-health approach in this study is justified because the traditional eco-health approach is criticized for overlooking institutional responses that shape forest-based communities’ day-to-day adaptive resource use behavior (Dakubo 2010; Harrison et al. 2019). The eco-health approach integrates variations in resource user groups’ experiences and coping strategies due to changing events, such as health shocks, which may shape or be shaped by forest management institutions (Dakubo 2010; Harrison et al. 2019). Captured as structures and processes by Fleetwood (2008), Kimengsi et al. (2022) noted that institutions in resource-dependent communities exhibit (in)formal typologies by structures (e.g., governmental organizations, NGOs, local cults, vigilante groups, etc.) and by processes (laws, rules, taboos, norms, etc.).

These institutions shape resource users’ demand, exploitation, and forest-linked livelihood strategies. On the other hand, institutions are reversely shaped by shock events, including health-related ones, as resource users adapt to their day-to-day realities. To examine how institutions evolve under health shocks, this study adopts Koning’s (2016) institutional change mechanisms. This framework is particularly apt, as it captures both gradual and abrupt changes in institutional forms and functions—providing a nuanced lens for understanding how forest-linked institutions adapt, recalibrate, or weaken in response to socio-ecological disruptions. As defined in Table 1, institutional change mechanisms include patch-up, transposition, puzzling, layering, etc (Koning 2016). These changes further shape resource use behaviors, leading to different outcomes. The outcome could be (a) ecological (positive/negative), e.g., increased or reduced logging, poaching, and mining in forest areas, directly impacting forest ecology (b) economic (positive/negative), e.g., increased or decreased income for resource-dependent households, local resource management groups, and forest departments, (c) political (positive/negative), e.g., inclusion and participation of resource-dependent communities in forest management decision-making and conservation initiatives and (d) social (positive/negative), e.g., increased or decreased unemployment, social capital destruction due to resource use, and management conflicts (Krott et al. 2014; Schusser et al. 2015).

**Table 1:** Institutional change mechanisms identified in the study and their manifestations

Mechanism	Manifestation
Transposition	In the context of institutional change, the structures within which institutions are embedded remain fairly stable but function differently as a response mechanism to change (Koning 2016).
Patch-up	A reactive response of introducing new institutions to adapt to new social, economic, political, and ecological disequilibrium conditions (Koning 2016).
Layering	Working around difficult-to-change institutions by adding different layers (institutions) to make the difficult-to-change institutions obsolete or malfunction (Koning 2016).
Exhaustion	When evolving events break down or make existing institutions obsolete. This can take the route of dismantling the institution or shut down functionally (Koning 2016).
Erosion	Gradual endogenous changes that undermine institutions with the potency of leading to exhaustion in the long term (Gerschewski 2021a, 2021b).
Puzzling	Recalibrating institutions in a trial-and-error fashion to weed out undesired implications (Koning 2016).
Expansion	It entails enforcing institutions beyond their coverage at the equilibria condition (Koning 2016).
Reinforcement	Stricter enforcement or more intensified rules or norms as a consequence of social events change (Genschel 1997).

### Data Collection Methods

**Fig. 2:**
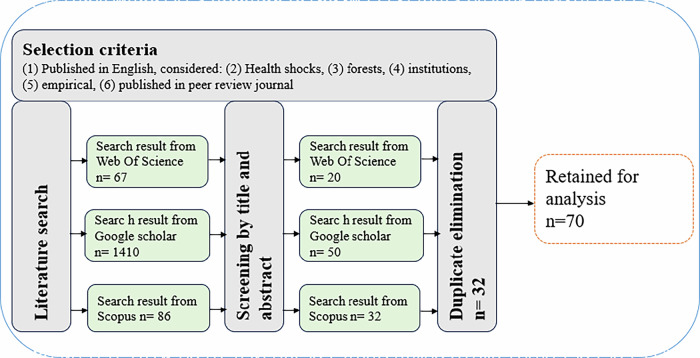
Systematic review protocol of how papers used for the analysis were sorted (Kimengsi et al. 2022; Mengist et al. 2020)

We adopted the systematic review protocols in sorting articles for this study (Kimengsi et al. 2022; Mengist et al. 2020). We first developed a list of search words covering “institutions,” “change,” “forest,” “management,” and “health shocks” as the thematic themes. See supplementary (SP) Table 1 in the Appendix. These search terms were combined using Boolean operators like “AND” or “OR” to develop search strings across three databases: (1) Web of Science, (2) Scopus, and (3) Google Scholar. We started our literature search from the Scopus database because it covers a broader range of publications and international journals, including comprehensive coverage of social sciences, humanities, and natural sciences (Gusenbauer 2020). Further, it gives powerful search capabilities, including advanced filtering options (ibid). An initial combination of all the words developed in SP Table 1 did not yield results; hence, we resorted to selecting search words carefully guided by the study objectives. This led to the first search string “Institution*” AND (“Change*” OR “Response*”) AND (“Outbreak*” OR “Health shock*” OR “health-related shock*” OR “COVID-19” OR “Ebola” OR “HIV” OR “Malaria”) AND (“Forest* OR Forest management*”). This string produced 86 articles. The limited nature of articles in the Scopus database necessitated further searching in the Web of Science database due to its robust coverage of journals in the field of natural sciences, which includes a selection of high-impact journals from various disciplines (Li et al. 2018). We applied the search string developed in the Scopus database to ensure uniformity, which found only 4 articles. Given this, we resorted to creating a new search string “Formal institution*” OR “Informal institution*” Or “Endogenous institution*” OR “Exogenous institution*” AND “Change*” OR “Shift*” OR “Modification*” OR “Alteration*” AND “Forest*” OR “Timber*” OR “NTFPs*” OR “Ecosystem*” AND “Management*” OR “Governance*” OR “Control*” OR “Access*” OR “Administration*” OR “Supervision*” OR “Stewardship*” OR “Operation*” AND “Pandemics*” OR “Endemic*” OR “Health shock*” OR “Health crises*” OR “Public health emergency*” OR “Disease*” OR “Outbreak*” OR “Spillover*.” This search string used in the Web of Science database produced 67 articles. Further, the Google Scholar database was employed due to its wide coverage. This was to increase our search and article selection variability. We repeated the search string used in Web of Science, but it produced many unrelated materials. We, therefore, applied the search string developed in the Scopus database, which produced 1410 articles. The articles found in all the databases were selected based on language -English, empirically conducted, published in peer-reviewed journals, considered forests, institutions, and finally, health shocks. These articles were screened for relevant articles (Fig. 2). Further, we copied all the articles chosen at the screening stage and arranged them alphabetically in a Word document. This allowed us to see duplicated materials and deleted them easily. In all, 70 articles were retained and used for the analysis. It should be noted that our search found articles starting from 2003. This is probably a result of the MDGs introduced in the year 2000, which streamlined health issues (MDG 1), poverty and hunger (MDG 1), and environmental sustainability (MDG 7), propelling multi and interdisciplinary research at the intersection of health and forest management.

### Data Analysis Methods

The abstract, methods, conclusion, and, in some cases, the results and the discussion sections of the articles sorted for analysis were read thoroughly to extract data for the study. This was guided by the analytical framework, which provided a clear focus for the study. We employed content analysis since health shocks, institutional change, and their outcomes are intricate and not often stated directly (Hsieh and Shannon 2005). In doing this, data were clustered under four major themes following the study objectives and the analytical framework: (1) *conceptualization of health shocks in the literature*, (2) *conceptualization of institutions and institutional change mechanisms*. (3) *management outcomes of forest-linked institutions shaped by health shocks*, and (4) *methodological approaches employed*. Key text with variable(s) of interest was highlighted, extracted, coded, and curated in an Excel spreadsheet following the study questions and corresponding themes. This was judged fit since employing software for data extraction and analysis may overlook salient details due to the complexity of the subject under investigation (Hsieh and Shannon 2005). It is worth noting that institutional change mechanisms were initially coded by the first author based on the conceptual definitions in Table 1. These classifications were then reviewed and refined collaboratively with the second and third authors to ensure consistency. Discrepancies were resolved through iterative discussions guided by the framework. Descriptive analysis further aided in establishing the regional variations in the conceptualization of health shocks, institutions, their change mechanism, outcomes, and the methodology employed so far. Further, key text extracted from the literature was used to justify and contextualize the descriptive analysis. We used the ArcMap^TM^ 10.2.2 version to report the global distribution of cases in the review.

## Results

### Global Trend and Distribution of Case Studies

**Fig. 3:**
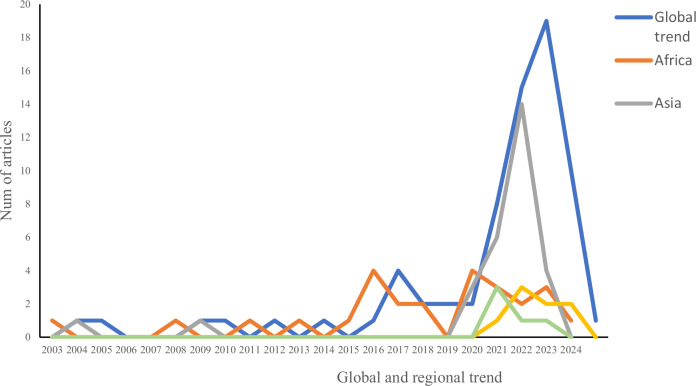
Evolution of the literature on health shocks and forest-linked institutions

The trend (Fig. 3) shows that health shocks and forest-linked institutional changes gained scholarly attention from 2003 until 2024, but with geographical and temporal variations. The global trend shows that between 2003 and 2015, academic attention on this topic was minimal, leading to a handful of publications worldwide. A gradual global increase in publications on health shocks and forest-linked institutional change started in 2016, particularly in Africa (*n* = 4). From 2016 to 2019, interest in the subject at the global level remained modest but with a study pattern. The global turning point was observed in 2020, possibly with the outbreak of the COVID-19 pandemic, which brought to attention its possible linkages with varying forest use behaviors that could shape forest-linked institutions. The year witnessed a considerable increase in publications on the subject (*n* = 8). This global trend intensified in 2021 (*n* = 15) and 2022 (*n* = 19), signaling an increased awareness and interest in how health shocks interact with forest governance systems through institutional change. Regionally, Asia contributed significantly to the subject’s publication surge (*n* = 29), closely followed by Africa (*n* = 28), Europe/North America (*n* = 8), and then Latin America (*n* = 5). See supplementary (SP) Table 2 for details.

**Fig. 4:**
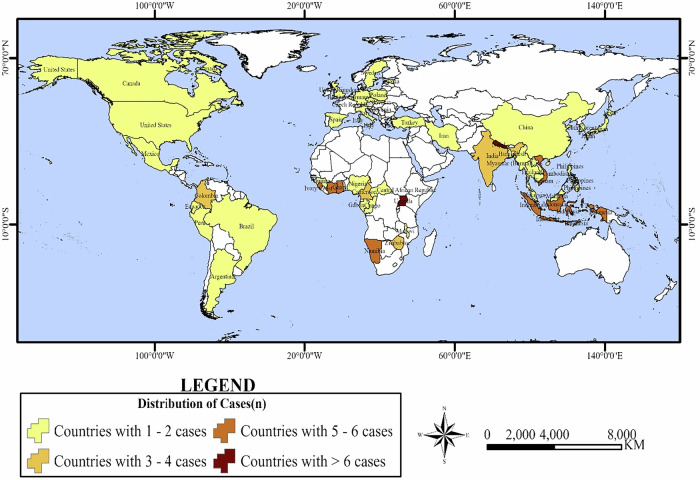
Global distribution of health shocks and forest-linked institution cases

On a case basis, the result shows that most of the 107 cases on the subject are from Africa (*n* = 44), followed by Asia (*n* = 39). Europe/North America followed with 17, and Latin America followed with 7. We further teased out the cases by countries (Fig. 4) and subregions (SP Table 2). The review unearths that most cases in Africa emanate from West Africa (*n* = 19), with Sierra Leone recording the highest number of cases (*n* = 6). However, country-level statistics in West Africa show gaps in exploring health shocks and forest-linked institutional change in Ghana, Nigeria, Guinea, and Ivory Coast (SP Table 2). Southern Africa came second (*n* = 9), with Namibia as the leading country, while Central and East Africa followed with eight cases each. Cameroon (*n* = 4) and Uganda (*n* = 7) are the leading countries in these subregions. The country-level data shows gaps exist in the DRC Congo, Gabon, and the Central African Republic. Southeast Asia (*n* = 19) recorded the most cases in Asia, with many cases reported in Indonesia (*n* = 6). However, countries including Cambodia (*n* = 1), Myanmar (*n* = 1), Philippines (*n* = 1), Thailand (*n* = 1), and Laos (*n* = 1) are significantly under-studied in terms of health shocks, forest-linked institutional change, and their possible outcomes. South Asia followed (*n* = 14), with Nepal recording nine cases, the highest, with gaps in Bangladesh (*n* = 1) and India (*n* = 4). East Asia and Western Asia were equalized, with each recording three cases. China, Japan, and the Republic of Korea recorded a case each, while Iran (*n* = 2) was the highest in Western Asia (SP Table 2). In the Europe/North America cluster, more cases were found in Europe (*n* = 11) than in North America (*n* = 6). By subregion, many cases were found in Western Europe (*n* = 4), Central Europe (*n* = 3), Northern (*n* = 2), and Eastern Europe (*n* = 2). In North America, Mexico and the United States reported two cases each, with only one from Canada. As a major forest player in the Boreal region, further studies on health shocks and how they (re)shape forest-linked institutions, especially among Indigenous tribes and their outcomes, should be conducted. In Latin America, 5 cases were found in the Andean region: Colombia (*n* = 3), Peru (*n* = 1), and Ecuador (*n* = 1). Only two cases were found in the Southern Cone, Brazil (*n* = 1) and Argentina (*n* = 1). This implies that more studies on the subject need to be conducted in these areas since they are significant players in tropical forest ecosystems (SP Table 2).

### Health Shocks and Forest-Linked Institutional Conceptualization in the Literature

A majority of the reviewed literature—more than half—conceptualizes health shocks as pandemics (e.g., COVID-19). About one-fourth of the studies interpret health shocks in terms of epidemics (e.g., Ebola), while fewer than 1 in 10 consider endemic framings (e.g., malaria), with regional variations. In Africa, nearly one in four studies emphasize epidemic framings, followed by just over 1 in 10 that highlight pandemics, and a small proportion focusing on endemics. In Asia, nearly one-third of the studies adopt a pandemic lens, with very few considering epidemics or endemics. A similar pandemic-centered pattern is evident in Europe and North America, where no studies conceptualize health shocks as epidemics or endemics. Likewise, in Latin America, a small but notable share of the literature frames health shocks as pandemics, with no records linking forest-linked institutional change to either epidemics or endemics (see Table 2 for absolute statistics).

**Table 2:** Absolute number and percentage of articles on health shock typology in forest-linked institution literature reviewed

Health shock typology	Africa (%)	Asia (%)	Europe and North America (%)	Latin America (%)	Total (%)
Pandemic	10 (13.9)	22 (30.6)	7 (9.2)	5 (6.9)	44 (61.0)
Endemic	3 (4.2)	3 (4.2)	0 (0.0)	0 (0.0)	6 (8.4)
Epidemic	16 (22.2)	3 (4.2)	0 (0.0)	0 (0.0)	19 (26.4)
Others	1 (1.4)	1 (1.4)	1 (1.4)	0 (0.0)	3 (4.2)
Total by continent	30 (41.7)	29 (40.4)	8 (10.6)	5 (6.9)	72 (100)

Two articles published in Africa considered the COVID-19 pandemic and Ebola as an epidemic, scaling up the number to 72 instead of 70

The literature largely conceptualizes forest-linked institutions as processes rather than structures, with formal and informal processes each representing nearly half of the reviewed studies. In contrast, formal structures appear in less than 1 in 10 studies, while informal structures are referenced in only a small fraction. Regionally, Asia contributes the largest share of studies framing institutions as formal processes—over one-fifth—followed by Africa with about one-seventh, Europe/North America with just under one-tenth, and Latin America with a small portion. Regarding informal processes, Africa clearly dominates, accounting for roughly one-fourth of the studies, while Asia contributes about one-seventh, with Europe/North America and Latin America each representing a very small share. In terms of structural dimensions, formal structures are referenced more frequently than informal ones, but still in less than 1 in 10 studies overall. Africa again leads in this category, contributing over half of the studies on formal structures, while Asia, Europe/North America, and Latin America each contribute only a small portion. When it comes to informal structures, only Africa and Asia make mention of this category, each accounting for a marginal share, while no studies from Europe/North America or Latin America report on informal structural framings (see Table 3 for absolute statistics).

**Table 3:** Absolute number and percentage of articles on the conceptualization of forest-linked institutions in the literature analyzing health shocks and institutions

World regions	Institutions as structures		Institutions as processes		Total
	Formal (%)	Informal (%)	Formal (%)	Informal (%)	
Africa	4 (4.7)	1 (1.2)	12 (14.1)	22 (25.9)	39 (45.9)
Asia	1 (1.2)	1 (1.2)	19 (22.4)	12 (14.1)	33 (38.9)
Europe and North America	1 (1.2)	0 (0.0)	5 (5.9)	3 (3.5)	9 (10.6)
Latin America	1 (1.2)	0 (0.0)	2 (2.4)	1 (1.2)	4 (4.7)
Total	7 (8.2)	2 (2.4)	38 (44.7)	38 (44.7)	85 (100)

*n* = 85 because some articles captured both formal and informal institutions (in terms of the structures and processes divide) in their analysis, while others only captured formal or informal processes

### Mechanisms of Formal and Informal Forest-Linked Institutional Change Induced by Health Shocks

#### Formal Institutional Change Mechanisms

The literature identifies patch-up as the most frequently reported mechanism of formal institutional change in response to health shocks, appearing in about two-fifths of the reviewed studies. Regionally, Asia leads in reporting this mechanism, contributing over one-sixth of the total literature, followed closely by Africa with just under one-sixth. Europe/North America and Latin America follow with smaller shares. Puzzling emerges as the second most common mechanism globally featured in roughly one-fourth of the studies, with notable regional differences. Asia alone accounts for more than 1 in 10 studies on puzzling, while Europe/North America contributes an equal share, and Africa accounts for a smaller portion. No puzzling mechanisms are reported in Latin America. Expansion of formal institutions is noted in about one in eight studies, with Africa contributing the largest share, just under 1 in 10—followed by Asia, which represents a small portion. Europe/North America and Latin America record no instances of this mechanism. Transposition appears in less than 1 in 10 studies, with Africa again leading, and smaller but equal contributions from Asia and Latin America. Both exhaustion and reinforcement are relatively rare, each featuring in just over one-twentieth of the literature. In terms of exhaustion, Africa, Asia, and Latin America each contribute a marginal share, while Europe/North America shows no evidence of it. Reinforcement is more commonly reported in Africa, with a small number of studies from Asia, and none from the other regions. Finally, institutional layering is the least documented mechanism, appearing in only a very small fraction of the literature and solely reported in Latin America (Table 4). See Table 5 for empirical examples of the institutional change mechanisms teased out in the literature.

**Table 4:** Absolute number and percentage of articles on health shocks and formal institutional change mechanisms

Mechanism	Africa (%)	Asia (%)	Europe/North America (%)	Latin America (%)	Case summation (%)
Patch-up	8 (14.5)	9 (16.4)	3 (5.5)	2 (3.6)	22 (40)
Expansion	5 (9.1)	2 (3.6)	0 (0.0)	0 (0.0)	7 (12.7)
Puzzling	3 (5.5)	8 (14.5)	3 (5.5)	0 (0.0)	14 (25.5)
Exhaustion	1 (1.8)	1 (1.8)	0 (0.0)	1 (1.8)	3 (5.4)
Erosion	0 (0.0)	0 (0.0)	0 (0.0)	0 (0.0)	0 (0.0)
Transposition	2 (3.6)	1 (1.8)	0 (0.0)	1 (1.8)	4 (7.2)
Reinforcement	2 (3.6)	1(1.8)	0 (0.0)	0 (0.0)	3 (5.4)
Layering	0 (0.0)	0 (0.0)	0 (0.0)	1 (1.8)	1 (1.8)
Total	23 (41.8)	21 (38.1)	6 (11)	5 (9.1)	55 (100)

**Table 5:** Extracts of formal institutional responses to health shocks from the literature

Mechanism	Response
Patch-up	Forest officers established informal, anonymous communication officers (village spies) in forest-based communities in Uganda (Acquaah et al. 2023, 2024).
	A total ban on hunting, trade, and consumption of stereotyped species, e.g., Pangolin, Chimpanzees, Bats, and monkeys, in China and Ivory Coast (Gossé et al. 2023; Xiao et al. 2021).
Expansion	No hunting in forest reserves was expanded to include non-reserved areas such as community forests and buffer zones during health shocks, e.g., Ebola and COVID-19 in Uganda (Acquaah et al. 2023).
	Medicinal plants that were believed to have curative properties against health shocks such as flu and fever were believed to function the same against COVID-19 and Ebola in Sierra Leon and Belgium (Bai et al. 2019; De Meyer et al. 2022).
Puzzling	Laying off forest management staff ban on recruiting and training new staff, as a litmus mechanism in Indonesia (Larasatie et al. 2022).
	Increased forest patrols as a litmus test mechanism in Uganda (Acquaah et al. 2023).
Exhaustion	Some forest departments forcefully shut down as a reactive incidence and prevalence response to health shocks, e.g., COVID-19 in Nepal (Maraseni et al. 2022).
Transposition	Forest patrol joined in community sensitization during health shocks, e.g., COVID-19 and Ebola in Zimbabwe (Ndlovu and Mjimba 2021).
	Tourist guards joined forest officers and community forest law enforcement officers in Ghana (Soliku et al. 2021).
Reinforcement	Bans on hunting, wildlife trade, and consumption were reinforced. For instance, there was a total ban on bushmeat trade and consumption due to the Ebola outbreak, leading to the police mounting several road checkpoints to limit the trade flow of wild meat across villages, districts, and regions in Sierra Leone (Gossé et al. 2023; Mufunda et al. 2016).
Layering	Deliberate enactment of policies making the pre-health shock (COVID-19) strict policies that limited forest resource extraction obsolete in Brazil (Vale et al. 2021).

#### Informal Institutional Change Mechanisms

The literature predominantly frames informal institutional change during health shocks through the mechanism of expansion, which accounts for just under two-fifths of all reviewed studies (Table 6). Regionally, Africa leads significantly, contributing over a quarter of the studies on informal institutional expansion, while Asia and Europe/North America each account for a small share. Patch-up follows as the second most reported mechanism, appearing in nearly one-third of the literature. Again, Africa dominates, contributing almost one-fifth of global studies, with Asia representing one-tenth and Latin America a very small share; Europe/North America, however, shows a noticeable gap. The transposition of informal institutions ranks third globally, featured in one-tenth of studies. Here, Asia leads, followed by Africa, while Europe/North America and Latin America are entirely unrepresented—indicating significant knowledge gaps. Puzzling is referenced in a smaller portion of the literature (under one-tenth), with Africa contributing the bulk and Asia a minor share, and once again, Europe/North America and Latin America remain absent. Roughly one in twenty studies report erosion of informal institutions, primarily from Asia and, to a lesser extent, Africa. Exhaustion and reinforcement each appear in only a small fraction of the literature (4%), with exhaustion reported solely in Asia, while all instances of reinforcement are from Africa. Notably, the layering mechanism is absent entirely from the informal institutional change discourse. See Table 7 for empirical examples of the institutional change mechanisms teased out from the literature.

**Table 6:** Absolute number and percentage of articles reporting health shocks and informal institutional change mechanisms

Mechanism	Africa (%)	Asia (%)	Europe/North America (%)	Latin America (%)	Case summation (%)
Expansion	13 (26.0)	3 (6.0)	3 (6.0)	0 (0.0)	19 (38.0)
Patch-up	9 (18.0)	5 (10.0)	0 (0.0)	1 (2.0)	15 (30.0)
Transposition	2 (4.0)	3 (6.0)	0 (0.0)	0 (0.0)	5 (10.0)
Puzzling	3 (6.0)	1 (2.0)	0 (0.0)	0 (0.0)	4 (8.0)
Erosion	1 (2.0)	2 (4.0)	0 (0.0)	0 (0.0)	3 (6.0)
Exhaustion	0 (0.0)	2 (4.0)	0 (0.0)	0 (0.0)	2 (4.0)
Reinforcement	2 (4.0)	0 (0.0)	0 (0.0)	0 (0.0)	2 (4.0)
Layering	0 (0.0)	0 (0.0)	0 (0.0)	0 (0.0)	0 (0.0)
Total	30 (60.0)	16 (32.0)	3 (6.0)	1 (2.0)	50 (100)

**Table 7:** Extract about informal institutional response to health shocks

Mechanism	Response
Patch-up	Community elders created a local sensitization group. Communities informally cautioned and banned members from hunting, eating, or going in close contact with such wildlife species in Uganda and Nigeria (Acquaah et al. 2023; Onyekuru et al. 2020).
	Women use HIV infection or the threat of it to challenge traditional norms and claim inheritance rights to the lands, including forested ones of their deceased husbands in Zambia (Emily and Unruh 2008).
Expansion	Communities expanded their beliefs in the preventive and curative properties of medicinal plants for the treatment of fever and influenza to include other health shocks such as COVID-19 and Ebola virus in Sierra Leone, Nepal, and Belgium (Bai et al. 2019; De Meyer et al. 2022; Khadka et al. 2021).
	Forests, previously perceived as places for relaxation and recreation, were also believed to have therapeutic benefits for mental health and boosting the immune system during health shocks in Germany and Slovakia (COVID-19) (Pichlerová et al. 2021).
Puzzling	Communities extracted and used previously medicinal plants with established curative and preventive for certain ailments against relatively new diseases as a litmus treatment in Guinea (Baldé et al. 2016).
Exhaustion	India recorded a collapse of local forest management groups due to economic and livelihood hardships imposed by health shocks, e.g., COVID-19 (Rana and Fleischman 2023).
Erosion	Community user group members reduced their commitment due to the ban on meetings, while others left the group searching for their livelihood due to a collapse of ecotourism activity in India (Rana and Fleischman 2023).
	Decay in the belief of traditional medicinal practices due to new dynamics introduced by the Ebola outbreak in Sierra Leone (Bai et al. 2019).

### Forest Management Outcomes Linked to Health-Shocks-Induced Institutional Change

Table 8 shows that a little over one-third of the articles reviewed connect changes in the forest-linked institutions studied in the context of health shocks with varying ecological outcomes. For instance, just over one-tenth link the institutional change studied in the context of health shocks with positive ecological outcomes, manifesting in reduced forest extraction activities such as (il)legal logging and poaching. However, those reporting on negative ecological outcomes, manifesting increased forest cover loss and decreased wildlife population due to increasing (il)legal logging and poaching, dominate the literature on ecological outcomes, accounting for just over one-quarter. Close to 3 in 10 of the articles reported on the economic outcomes of forest-linked institutional change in the context of health shocks, but with significant variations. Economic negative outcomes, such as reduced income, dominated those who reported on economic outcomes of the institutional change studied, as opposed to those reporting on positive economic outcomes (just over one-quarter vs less than one-thirtieth). This is followed by articles reporting the social outcomes of forest-linked institutional change induced by health shocks (again, close to three in ten). About one-quarter linked health shocks-induced institutional change with negative social outcomes, such as conflicts and social network deterioration, while just above one-twentieth linked these institutional changes with positive social outcomes. Lastly, only a small fraction of the literature reviewed reported on the political outcomes of health shocks-induced forest-linked institutional change. Positive political outcomes dominate in this literature segment, as do negative political outcomes. See Table 9 for empirical examples of the linked outcomes of forest-linked institutional change induced by health shocks. See Table 9 for empirical examples of the linked outcomes of forest-linked institutional change induced by health shocks.

**Table 8:** Absolute number and percentage of articles reporting the outcomes of institutional change shaped by health shocks

Outcomes	Direction of outcome	Number of reported cases by region					
		Africa (%)	Asia (%)	Europe/North America (%)	Latin America (%)	Total (%)	Case summation (%)
Ecological	Ecological (+)	1(1.4)	7 (9.7)	0 (0.0)	0 (0.0)	8 (11.2)	27 (37.5)
	Ecological (−)	6 (8.3)	10 (13.9)	1 (1.4)	2 (2.8)	19 (26.4)	
Economic	Economic (+)	0 (0.0)	2 (2.8)	0 (0.0)	0 (0.0)	2 (2.8)	21(29.2)
	Economic (−)	7 (9.7)	8 (11.2)	4 (5.6)	0 (0.0)	19 (26.4)	
Social	Social (+)	0 (0.0)	4 (5.6)	0 (0.0)	0 (0.0)	4 (5.6)	21(29.2)
	Social (−)	5 (6.9)	10 (13.9)	2 (2.8)	0 (0.0)	17 (23.6)	
Political	Political (+)	0 (0.0)	2 (2.8)	0 (0.0)	0 (0.0)	2 (2.8)	3 (4.2)
	Political (−)	0 (0.0)	0 (0.0)	0 (0.0)	1 (1.4)	1 (1.4)	

Ecological (+) = ecological positive; Ecological (−) = ecological negative; Economic (+) = economic positive; Economic (−) = economic negative; Political (+) = political positive; political (−) = political negative; Social (+) = social positive; Social (−) = social negative

**Table 9:** Extracts of the outcomes of institutions shaped by health shocks

Outcome	Extracts
Ecological (+)	With the closure of the Park, more staff were recalled from tour duties to law enforcement, which helped reduce illegal activities in Ghana (Soliku et al. 2021). Communities collaborated to fight illegal logging in Myanmar (Sapkota et al. 2022). The lockdown benefited Nepal’s wildlife movement and population (Koju et al. 2021). Legal frameworks protecting wildlife were strengthened in Vietnam, drastically reducing poaching (Pham et al. 2022), and forest fires were tremendously reduced in Indonesia (Behera et al. 2022).
Ecological (−)	Wildlife poaching and pressure on forest resource exploitation increased, including illegal fishing in mangroves, illegal logging and mining in conservation areas due to the absence of forest rangers and forest law enforcement officers in Zimbabwe, Nepal (Ndlovu et al. 2021), India (Rana and Fleischman 2023) and Iran (Rahimian et al. 2022). Increased deforestation in Colombia (Amador-Jiménez and Millner 2021) and Brazil (Vale et al. 2021).
Economic (+)	Rules mandating all forest-related employment to constitute 30% of community members of activity sites in Malaysia, leading to increased income (Atin and Lintangah 2023).
Economic (−)	Forest departments and communities lose income due to a decrease in ecotourism activities and park revenue in Zimbabwe (Ndlovu and Mjimba 2021) and Nepal (Maraseni et al. 2022). Decrease in bushmeat sales and market access difficulties in Ivory Coast (Gossé et al. 2023).
Political (+)	Increased community participation in forest management as communities collectively patrol forests in Malaysia and increased women’s participation in forest management during COVID-19 in Myanmar (Atin and Lintangah 2023; Sapkota et al. 2022).
Political (−)	Alienation of indigenous forest user groups from forest access/use and participation in forest management in Colombia (Amador-Jiménez and Millner 2021).
Social (+)	Resource (money) circulation/increased benefit sharing and social reciprocities among community forest members in Cambodia, Myanmar, Vietnam, and Nepal (Gentle et al. 2020; Sapkota et al. 2022) and capacity development in Malaysia (Atin and Lintangah 2023).
Social (−)	Obstructed social networks (capital) of ecotourism communities with people from other countries in Ghana (Soliku et al. 2021). Increased unemployment and customary ostracism in Sierra Leone, Ghana (Goguen and Bolten 2017). Destroyed social networks between some community members who beat up forest officials in India (Rana and Fleischman 2023). Marrying of young children to reduce economic pressure in Bangladesh (Hossain et al. 2022). Overcrowding due to high forest visitation leads to conflict between user groups in the UK, Germany, and Spain (Mcginlay et al. 2020).

### Methodologies Employed in Analyzing Forest-Linked Institutions Shaped by Health Shocks

Table 10 shows that qualitative methods are the most prioritized methodology for investigating health shocks-induced forest-linked institutional change and their linked forest management outcomes—used in just under half of the studies reviewed. Regionally, investigations from Asia dominate qualitative methods, representing just over one-fifth, followed closely by Africa at one-fifth, while only a small fraction of the literature from Europe/North America and Latin America is solely based on qualitative investigation. Qualitative methods are followed by a combination of qualitative and quantitative methods (mixed method) used in just over one-quarter of the literature. Literature from Asia again leads in the use of mixed methods at just under one-tenth, followed by Africa at one-tenth, Europe/North America at just over one-twentieth, and Latin America at a small fraction. The use of multi-methods—reported in just under one-fifth of the studies—followed a similar regional trend: Asia again led with just under one-tenth, followed by Africa at just over one-twentieth, Europe/North America at a small fraction, and Latin America at a marginal share. The use of the sole quantitative method is the least prioritized methodology for investigating health shocks-induced forest-linked institutional change and their linked forest management outcomes, appearing in only 1 in 10 studies. African literature dominates this category, contributing just under one-fifteenth, followed by Asia at a small fraction and Europe/North America at a marginal share, with no instance of sole quantitative methods found in Latin American literature. Regarding case studies, nearly three-quarters of the literature employs a single-case approach. Of this, roughly one-third of the literature from Asia, just over one-quarter from Africa, one-tenth from Europe/North America, and a small fraction from Latin America employed a single-case approach for their investigations. The multiple-case approach is used in just over one-quarter of the studies we examined. Africa dominates the use of multiple-case studies with just under one-seventh, followed by Asia at just under one-tenth, Europe/North America at just over one-twentieth, while no literature from Latin America employed a multiple-case approach in their investigations.

**Table 10:** Absolute number and percentage of articles on the various methodologies employed so far

Methodology	Africa	Asia	Europe and North America	Latin America	Total
Qualitative	14 (20.0)	15 (21.4)	2 (2.9)	2 (2.9)	33 (47.1)
Mixed method	7 (10.0)	6 (8.6)	3 (4.3)	2 (2.9)	18 (25.7)
Multimethod	3 (4.3)	6 (8.6)	2 (2.9)	1 (1.4)	12 (17.1)
Quantitative	4 (5.7)	2 (2.9)	1 (1.4)	0 (0.0)	7 (10.0)
Total	28 (40)	29 (41.5)	8 (11.4)	5 (7.1)	70 (100)
Single-case approach	19 (27.1)	23 (32.9)	7 (10.0)	3 (4.3)	52 (74.3)
Multiple-case approach	9 (12.9)	6 (8.6)	3 (4.3)	0 (0.0)	18 (25.7)
Total	28 (40)	29 (41.4)	10 (14.3)	3 (4.3)	70 (100)

## Discussion

### Health Shocks and Forest-Linked Institutional Change Research Trend

The results show a global surge of publications on health shocks and forest-linked institutional change in 2020. With the advent of COVID-19 in 2019, the surge in publications regarding the topic under investigation in this period is not surprising. A possible explanation is that the COVID-19 pandemic transcended the realms of significant health shocks by triggering global macroeconomic downturns (Aktar et al. 2021). This may have triggered interest in how forest-linked institutions are reshaped since forests function as economic and livelihood buffers during hard times (Kuuwill et al. 2022). Regionally, the study results reveal a significant publication surge in Asia and Africa. Unsurprisingly, these are tropical regions well-noted for a significant umbilical connection between forests, livelihoods, and well-being (Davis et al. 2025; Sebego et al. 2019; Kuuwill et al. 2022). Furthermore, studies over the years have characterized Africa and Asia as regions with frequent health shocks (Barbiero 2020; Gully 2020). This may have already attracted interest in the subject, with COVID-19 rekindling such interest. In Asia, for instance, the position of Southeast Asia could thus partly be related to the region’s landscape, which has dense forests, a variety of ecosystem types, and forest reliance (Prothero 1999). This could also explain Nepal’s position in South Asia, given the country’s high reliance on forests and its merits as a pacesetter in community forestry (Kimengsi and Bhusal 2022). This may have drawn considerable interest in research into how health shocks like COVID-19 affect forest-linked institutions and their implications for forest management.

In the context of Africa, the subject may have already gained some level of research attention due to its severe incidence of health shocks, such as Ebola, HIV, and Mpox, with weak healthcare systems (Barbiero 2020). Evidence of this in countries such as Namibia, Cameroon, Uganda, and Sierra Leon, among others, possibly reflects health shock vulnerabilities due to environmental and socioeconomic reasons and weak institutional arrangements that perpetuate health shocks. The limited scientific interest of the subject in Europe/North America over the years is possibly due to robust forest management institutions coupled with limited direct forest dependencies. Hence, it is reasonable for the results to reveal publication interest in the subject from 2022, possibly because of the evidence of COVID-19 (re)shaping forest visits and other forest use perceptions (Derks et al. 2020). Latin America recorded the least publications, which is surprising given empirical evidence of forest reliance in this region, similar to Africa and Asia (Brancalion et al. 2020). Nevertheless, we acknowledge that this is likely due to excluding publications not written in English. Also, this may partly be a factor of the complicated socio-political landscape, where land tenure conflicts are more widespread and studied in relation to institutional change than health shocks. The global and regional trend reveals growing but relatively sparse scholarly engagement. Despite limited evidence, the pattern suggests a significant but short-lived spike in scholasticism of the topic under investigation.

### Forest-Linked Institutions and Health Shocks Conceptualization

The findings reveal a strong thematic concentration on pandemics within the literature addressing forest-linked institutional change in the context of health shocks in Asia. This follows the conventional knowledge that COVID-19 started in Asia and was framed as a zoonotic outbreak, leading to institutional change in various facets of the region, including the forest sector (Kumar et al. 2020; Tiwari et al. 2020). It is also not surprising that health shocks are conceptualized as pandemics in the reviewed literature in these two regions in Europe/North America, since COVID-19 reportedly impacted these regions significantly (Pachetti et al. 2020). A similar pattern is observed in Latin America, indicating that the literature in this regard has critically overlooked regional-level crises that may also drive institutional change. Such limitations hinder global efforts toward building resilient societies that are resilient to health shocks while achieving forest sustainability around tropical regions. However, epidemics dominate health shock conceptualization in Africa in relation to forest-linked institutional change research. This indicates that forest-linked institutional change is centered mainly on major regional health crises—such as Ebola and monkeypox—which have disproportionately impacted Africa and are often considered to have originated there (Adokiya and Awoonor-Williams 2016; Anuradha and Rao 2023).

However, the limited comparative evidence on health shocks and forest-linked institutional change in the context of global health shocks (e.g., COVID-19) and regional ones (e.g., Ebola) could undermine health shocks surveillance and forest sustainability efforts in the region. The review reveals that institutions are significantly conceptualized as processes compared with structures in the context of health shocks. This is unsurprising since shocks, including health-related ones, usually trigger different forest access behaviors and institutional responses (Acquaah et al. 2023). This possibly drives changes in the processes dimension as a temporary measure to accommodate such dynamics before complementing such changes with the structural institutional dimension. The high conceptualization of institutions as formal processes in Asia is likely due to the region’s political system, where formal structure, laws, and rules, among others, govern natural resource exploitation, including forests. In Africa, formal processes are largely rooted in the colonial hangovers shaping forest access and use (Kimengsi and Balgah 2021). However, the dominance of informal processes conceptualization in the African literature corroborates the argument that informal institutions primarily characterize forest access and use in the region (Das 2022; Yeboah-Assiamah et al. 2019). This may have contributed to the increased scientific traction on the informal processes’ institutional dimension in contemporary literature examining health shocks and forest-linked institutional change. Contradictorily, African literature reports more on the formal structures dimension of institutions relative to other regions. This agrees with previous studies reporting that formal structures dominate informal structures in resource governance in Africa despite the continent being characterized by informal arrangements (Kimengsi et al. 2023).

The dominance of formal structures in African institutions is representative of the emphasis on tangible frameworks due to the states’ and NGOs’ centralization of forest management. However, the limited focus on informal structures from their own praxis creates a need to investigate their responses to health shocks. A similar pattern is observed in Asia, where informal forest-linked institutions are sparsely reported in relation to formal ones. Europe/North America and Latin America did not report on informal institutions in the context of health shocks. This suggests that informal institutions, despite their prevalence in forest management, have been overshadowed by formal ones, resulting in limited research attention. In a nutshell, the dynamics of formal institutions (structures and processes) induced by health shocks are reported more than informal institutions (structures and processes). This validates the need for continuous studies on health shocks and forest-linked institutional change in the tropics, with a key focus on informal structures and processes. However, complementing such studies with formal structures and processes could further foster a holistic perspective on the subject.

### Formal Institutional Change Mechanisms in the Context of Health Shocks

The review results show changes in formal forest-linked institutions through diverse mechanisms in the context of health shocks. This indicates that health shocks induce diverse forest resource use behaviors, which in turn shape institutions regulating their access and use. This agrees with the historical institutionalist school of thought, particularly the punctuated equilibrium/path dependence model, which argues that institutions change abruptly due to external shocks (Gerschewski 2021b). For instance, the results show patch-up, defined as the reactive response of introducing new institutions to adapt to new social dynamics (Koning, 2016), as the dominant institutional change mechanism. Unsurprisingly, institutional change in Asia and Africa dominantly depicts this mechanism. This could be explained by the reactive response of governments in these regions to control extensive forest use to remediate the socioeconomic hardships of health shocks such as the COVID-19 pandemic. This agrees with the literature argument that the livelihoods of most households in Asia and Africa hinge on the forest (Kuuwill et al. 2022; Newton et al. 2016). Hence, reverse migration to rural areas during COVID-19 may have placed a high demand on the forest, leading to institutional patch-ups. This also aligns with empirical reports in Uganda, where forest officers responsively established anonymous village communication representatives for prompt information regarding illegal forest extraction during the COVID-19 pandemic (Acquaah et al. 2024). Other evidence suggests cases where the zoonotic narratives around health shocks like Ebola and COVID-19 resulted in the introduction of reactive formal institutions banning hunting, wildlife trade, and consumption in China and the Ivory Coast (Gossé et al. 2023; Xiao et al. 2021). This closely ties in with puzzling, where institutions are purposely recalibrated in trial and error to weed out undesirable consequences (Koning, 2016). The dominance of this mechanism in the Asian and African institutional change mechanism suggests that most forest management departments in the global south depend on income from foreign donors and internally through ecotourism activities. Hence, global shocks (e.g., pandemics) that limit forest-related external investment while reducing ecotourism activities destabilize forest management in these tropical regions. This largely leads to institutional puzzling, exemplified by temporarily laying off and banning the recruitment of forest workers (Larasatie et al. 2022). This may have also resulted in exhaustion, typifying the collapse of some formal institutions in Asia, Africa, and Latin America. Hence, there is a need for diversifying forest funding mechanisms to reduce dependency on external funding and ecotourism in the global south.

This can enhance institutional resilience during global shocks. In Europe/North America, puzzling epitomizes the temporary closure of open forest spaces as a litmus health shock management mechanism due to the visitor boom during the COVID-19 pandemic (Derks et al. 2020; Mcginlay et al. 2020). Also, the results indicate that expansion as an institutional change mechanism dominated in Africa, followed by Asia. This manifested in extending restrictions on forest reserve extraction to include off-reserve areas such as buffer zones and private forests (Acquaah et al. 2023). From a managerial perspective, we submit that expansion may have been ostensibly used under the need to address public health concerns, but with the prime motive to serve implicit conservation initiatives in practice. The limited record of this in Latin America is probably due to under-representation based on our exclusion criteria or a general lack of evidence. Another key institutional change mechanism recorded in the context of health shocks is the transposition of formal structures to new functions in Africa and Latin America. A possible explanation for this is that Africa, like Latin America, is traditionally known for financial, technical, and logistical challenges. Hence, transposition may have been dominantly used in these two regions as a feasible way of minimizing operational costs and procedural hurdles. In Ghana, for instance, the military and police, in charge of national and international security issues, assume new roles in helping forest patrol officers fight against illegal mining and other forest activities during the COVID-19 pandemic (Soliku et al. 2021). This suggests the need for developing regions to strengthen inter-agency collaboration frameworks in forest management to maximize existing institutional capacities and minimize operational costs during shocks, including health-related ones.

Also, old regulations limiting forest access, hunting, and consumption of zoonotic stereotyped species were reinforced, primarily in Africa. We submit that Ebola in Africa and COVID-19 in Asia propagated as zoonotic may have led to the strict application of institutions around hunting, trade, and consumption of some species in some parts of Africa. For instance, empirical reports in the Ivory Coast and Sierra Leone show that police checkpoints were mounted at various commercial routes to restrict passengers traveling with wild meat due to the Ebola and COVID-19 zoonotic narrative (Gossé et al. 2023). Lastly, we observed institutional layering, which is a deliberate attempt to make existing institutions impotent in Latin America. The literature reports cases where robust forest-linked institutions were layered to permit forest extraction in conservation areas in Brazil (Vale et al. 2021). This suggests that without significant decoupling of tropical livelihoods and economies from their strong link with the extractive industry, shocks will trigger institutional layering, leading to unsustainable extraction, as shown in the case of the COVID-19 pandemic.

### Forest Management Outcomes Associated with Health Shocks-Induced Forest-Linked Institutional Change

Our review results show that forest-linked institutional change in the context of health shocks is reportedly linked with diverse ecological, economic, social, and political forest management outcomes. It is possible that the reinforcement of formal institutions, expansion of existing ones, and institutional patch-ups seeking to limit forest access, especially during the COVID-19 pandemic, may have contributed to the positive ecological outcomes reported in the literature. Evidence of this exists in Uganda, where formal rules prohibiting forest extraction in forest reserves were extended to off-reserves, reducing forest extraction and poaching (Acquaah et al. 2024). The transposition of the military and ecotourism guards into forest patrol officers may have also contributed to reducing illegal logging and poaching (Soliku et al. 2021). In Asia, studies reported an increase in wildlife population and reduced forest extraction in Nepal, Vietnam, and Malaysia (Koju et al. 2021; Pham et al. 2022). This is possibly due to intensified forest monitoring (reinforcement) during the COVID-19 pandemic. Furthermore, the creation of informal community forest monitoring gangs (patch-up) during the health shocks could have further reinforced forest surveillance, possibly contributing to positive ecological outcomes. However, negative ecological outcomes linking health shocks-induced institutional change dominate the literature. A possible reason is the exhaustion and erosion of formal and informal institutions in the context of health shocks. Studies noted bans on recruiting forest staff, while some were temporarily relieved of their duties due to limited funds to cater for operational costs, as health shocks interrupted ecotourism and external funds.

This may have created operational gaps, which may have been exploited for increased logging and poaching. Evidence exists in India, where a small number of forest officers who confronted community members illegally extracting timber in the forest during the COVID-19 lockdown were beaten up (Rana and Fleischman 2023). This likely shocked the rest of the forest officers during this period as community members maximized the forest to abate economic hardships. Studies also report the disintegration of informal forest monitoring groups in Nepal due to health shock containment measures, resulting in a “gate-free” forest where everyone maximizes their benefits (Laudari et al. 2021). This mix of scientific evidence validates the need for a continuous investigation in Asia, where community forestry has gained prominence, in Africa, where 80% of its population depends on the forest, and in Latin America, which is significantly under-explored (Bijaya et al. 2016; FAO and UNEP 2020).

The literature also reports mixed economic outcomes connected to forest-linked institutional change induced by health shocks. In this regard, Asia dominated the reporting of positive economic forest management outcomes concerning forest-linked institutional change induced by health shocks. Evidence from Malaysia, for instance, suggests that the government introduced new regulations (patch-up) to reinforce existing ones during the COVID-19 pandemic, mandating forest extraction companies to allocate 30% of their total employment slots to local communities (Atin and Lintangah 2023). This was expected to contribute towards increasing the income of forest-based communities, a strategy to reduce illegal direct forest reliance around fringe communities amidst health shocks. This implies that patching up and reinforcing institutions that focus on providing alternative income earning in forest-dependent communities can positively affect forest resource sustainability. Nevertheless, negative economic outcomes were predominantly reported in some parts of Asia, followed by Africa, and then Europe/North America.

For example, forest departments and rural communities in Asia and Africa recorded income reduction due to market challenges and reduced income from national parks, ecotourism, and forest-related foreign direct investments during the COVID-19 pandemic (Gossé et al. 2023; Xiao et al. 2021). We argue that these negative economic outcomes may also account for the negative ecological outcomes, as the two are intricately linked. This reinforces our earlier position on diversifying income portfolios to ensure continuous financing of forest management operational costs in the global south. This can contribute to improving forest resilience amidst shocks. Decreased park revenues were also reported in the United States of America as the major negative economic outcome in connection with forest-linked institutions shaped by health shocks (Miller-Rushing et al. 2021). However, the economic outcomes of forest-linked institutions change induced by health shocks are less reported in Latin America.

The social outcomes of forest management linking institutions shaped by health shocks are significant in Asia and Africa, with Asia recording positive and negative social outcomes. In contrast, Africa and the remaining regions recorded only negative social outcomes. On the positive outcome, Cambodia, Myanmar, Vietnam, and Nepal report the social intervention of community forestry groups (Gentle et al. 2020; Sapkota et al. 2022). The accounts posit that community forest groups in these countries assumed new functions (transposition) by sensitizing their societies against health shocks (e.g., COVID-19). They also gave out their offices to contain affected persons for treatment and shared resources, including money, to help their communities. This flags the importance of community forestry in building resilient communities amidst health shocks.

Although community forestry has been introduced in Africa for nearly three decades, there is limited evidence of its social benefits to members and their communities during challenging times, such as health shocks. This calls for a close scientific investigation of the nature of community forestry practice in Africa, its benefit-sharing mechanisms, and social benefits during shocks. Regarding the negative social outcomes, many studies report rapid erosion of social capital, such as resource sharing between households. This is possibly because the expansion of conservation regulations to include off-reserve and the reinforcement of restrictions on forest extraction created constraints during health shocks, exemplified by COVID-19. Hence, many households may have limited resource sharing due to resource constraints, contradicting the traditional arguments of supportive social capital during hard times in rural societies (Kuuwill et al. 2024; Paumgarten et al. 2020). Expanding reserve regulation to cover off-reserves possibly also contributed to the reported heightened rural tension and conflicts between communities and forest managers in the context of health shocks (Rana and Fleischman 2023; Acquaah et al. 2023).

In Bangladesh, the change in institutions restricting forest access (patch-up) and its reinforcement contributed to intensifying household predicaments. In response, it is reported that most rural households ended up marrying off their underaged daughters to reduce the burden of providing for them (Hossain et al. 2022). It is also reported in Ghana that social and cultural displays around ecotourism sites got interrupted due to a complete shut down of ecotourism activities (patch-up). Although these social outcomes are reported, what is missing is the impact of these social outcomes on current or future community resilience and their implication for resource use and sustainability. In Europe/North America, reports from countries such as the United Kingdom, Germany, and Spain recounted significant competition, leading to conflict among users who visited forest areas during the COVID-19 pandemic (Mcginlay et al. 2020). This is likely linked to the expansion of resource users’ beliefs about forest environments’ therapeutic and immune-boosting properties in fighting the COVID-19 pandemic (Pichlerová et al. 2021).

Lastly, some Asian studies, especially in Malaysia and Myanmar, showed increased community participation in forest management as a positive political outcome linking forest-linked institutional change induced by health shocks (Atin and Lintangah 2023). This is not surprising based on the report that community forestry groups gave their offices as treatment centers for patients and supported constrained members financially in these countries. This may have exposed and reinforced the importance of forestry and belonging to community forest groups, resulting in increased participation in community forest management and decision-making. In effect, this study underlines that the involvement of forest fringe communities is a necessity insofar as forest sustainability during shocks is concerned. Studies reported that there has been exhaustion of some forest user groups, erosion of some informal ones, and alienation of indigenous forest user groups from forest management in Colombia within the context of health shocks (Amador-Jiménez and Millner 2021). However, we would like to submit that the political consequences of health shock-shaped forest-linked institutions are generally under-explored across the globe and hence call for scientific scrutiny.

### Methodologies Employed and Gaps

The review reveals a firm global reliance on qualitative methods in studying forest-linked institutional change in the context of health shocks and associated forest management outcomes. Asia and Africa are noted as heterogeneous cultural societies coupled with diverse forest-based dependencies and diverse formal and complex informal institutions regulating dynamic forest use behaviors (Kimengsi et al. 2023; Saikia and Mahanta 2025). Hence, this significant tilt toward qualitative methodology might be precipitated by the need for in-depth assessment and context-specific evidence regarding the topic. This supports the view that qualitative methodologies effectively capture in-depth insights into respondents’ experiences, perspectives, and interpretations—particularly valuable for exploring complex, multidisciplinary issues (AlQhtani 2025). This makes qualitative methodology suitable and handy for investigating topics like institutional change and their linked outcomes in the context of health shocks.

Nevertheless, the limited use of quantitative methodologies in this research stream constrains the ability to establish causality and assess the magnitude of institutional changes. This under-representation creates a methodological imbalance, as the strong reliance on qualitative approaches—while contextually rich—limits the generalizability, comparative strength, and causal attribution of findings. Therefore, we argue that a mixed-methods approach can address the respective limitations of purely qualitative or quantitative designs, enabling more robust, context-sensitive evidence and stronger analytical inference. Furthermore, the use of multi-methods represents a methodological potential that can be explored. The results also reveal an overwhelming reliance on single-case approaches globally. While useful for deep, context-specific understanding, this pattern limits the capacity to draw broader, comparative conclusions. The global methodological landscape of health shocks and forest-linked institutional change is highly skewed toward qualitative and single-case designs, constraining scalability and external validity.

## Review Limitations

This systematic review is limited to literature searches from Scopus, Web of Science, and Google Scholar. The authors acknowledge that other studies indexed in different databases, such as PubMed, ProQuest, JSTOR, etc., could have provided useful information for the study. Further, the review is limited to peer-reviewed articles published in English. This poses a limitation since other non-peer-reviewed materials published in other languages, such as Swahili, German, Dutch, Spanish, and French, among others, may have contributed to the review findings. This may have contributed to the limited studies from Latin America. While future studies should employ different databases in literature search, materials from other languages besides English should also be considered. We also focused on how health shocks shape forest-linked institutions without explicitly focusing on power features and actors’ interests. Since institutions do not operate in isolation (Giessen et al. 2014; Kimengsi et al. 2022), future studies should draw from actor-centered analysis and power theories (e.g., Krott et al. 2014) since actors may have engaged in the institutional change process informed by their interest. Also, the literature search ended in the first quarter of 2024, which is the extent to which the search could go at the time of the study in March 2024. Literature after this period, which may have probably influenced the review results, was not considered. Institutions are also conceptualized as (in)formal exogenous or endogenous dimensions, which are not captured in this study due to the quantum of information we presented. Hence, further research could unentangle this to inform policy. Also, institutional change in the context of climate change could further introduce nuances that can critically inform policies on resilient societies and forest management. We also acknowledge that institutional change does not always come about due to shocks, as examined in this study. Some evolve naturally (incrementally); research into institutions’ natural or incremental evolution can help unpack a holistic picture. Lastly, some of the institutional change mechanisms, such as puzzling and patch-up, seem to overlap conceptually.

## Conclusions and Future Research Pathways

This review reveals the dynamic nature of forest-linked institutional responses to health shocks and shows that regional variation is very important in shaping such responses. Drawing from the insights gained from this review, the following are the main conclusions derived: Firstly, the growing trend of research studies on forest-linked institutions shaped by health, especially via outbreaks of the Ebola virus and COVID-19, to heightened awareness of severe impacts on global and national, and resource-dependent economies. Our analysis shows that Asia emerged as the epicenter of literature on this topic, closely followed by Africa, Europe/North America, and Latin America. Regional differences in the conceptualization of health shocks, ranging from pandemics in Asia to epidemics in Africa, reflect the scale and intensity of human-forest interactions, with pandemics likely to emerge from Asia, while endemics mostly likely emerge from Africa. Secondly, most literature conceptualized institutions as processes rather than structures in analyzing health shocks-induced institutional change.

While informal processes dominate Africa, formal processes are more common in Asia, Europe/North America, and Latin America. Thirdly, our analysis of the mechanisms of institutional change is dominated by patch-up strategies followed by expansion and puzzling, calling for the need to understand diverse governance mechanisms and decision-making processes while analyzing the institutional responses to health shocks. While these institutional changes induced by health shocks led to some positive ecological and economic outcomes in Asia, the negative outcomes of institutional change were more pronounced than the positive, particularly in Asia and Africa. This highlights the need for proactive measures to mitigate adverse effects and capitalize on opportunities for enhancing resilience. Fourthly, the review highlights the importance of adopting mixed methods in analyzing health shocks and forest management institutions. Methodologically, the single-case study is prevalently adopted, indicating comparative methodological gaps in understanding the topic across different spatial and temporal scales.

Further empirical research is needed to deepen our understanding of health shocks-induced institutional dynamics and inform evidence-based policies that promote inclusive and sustainable forest management practices worldwide. Disciplines-specific research needs to be promoted regarding health shocks and institutional change, with a critical focus on power dynamics and political process. Specifically in the field of forestry, to prevent or deal with health shocks and their impact on forest sustainability. Also, forest governance research should investigate actors and their interests and power features in shaping forest-linked institutions during health shocks, which is significantly under-studied in the literature. Also, studying compliance with institutions shaped by health shocks is needed for building a resilient forest-linked institutional framework. Clinical research needs to pay more attention to the health preventive and curative properties of forest products in the different regions of the world to help combat health shocks and prepare societies for possible future shocks, which are predicted to be worse than what the world has witnessed already.

## Supplementary information

Appendix

## Data Availability

Data are provided within the Supplementary Information.

## Footnotes

1 Endemics are infectious diseases that are consistently present in a particular geographic area or population group at a relatively stable and predictable level (Morens et al. 2009). Endemics can persist for a long time without causing a wild outbreak.

2 An epidemic represents a rapid/sudden widespread disease occurrence within a particular geographic area or groups of people at different geographical locations. Epidemics can occur when a disease outbreak suddenly and affects a large number of people within a relatively short period.

3 Pandemics signify a disease that has spread across all continents, affecting many people (Morens et al. 2009).

## References

[CR1] Acquaah G, Kimengsi JN, Kuuwill A, Were AN (2024) Forest users’ perceptions and institutional dynamics during covariate health-related shocks: lessons from the Busitema Forest Reserve in Uganda. Sci Afr 24:e02134

[CR2] Acquaah G, Kimengsi JN, Were AN (2023) Response of forest management institutions to health-related shocks. Learning from the Busitema Forest Reserve of Uganda during the COVID-19 outbreak. For Trees Livelihoods, 32(3):167–188

[CR3] Adokiya MN, Awoonor-Williams JK (2016) Ebola virus disease surveillance and response preparedness in northern Ghana. Glob Health Action 9(1):29763. 27146443 10.3402/gha.v9.29763 PMC4856840

[CR4] Aduhene DT, Osei-Assibey E (2021) Socioeconomic impact of COVID-19 on Ghana’s economy: challenges and prospects. Int J Soc Econ 48(4):543–556.

[CR5] Aktar MA, Alam MM, Al-Amin AQ (2021) Global economic crisis, energy use, CO2 emissions, and policy roadmap amid COVID-19. Sustain Prod Consum 26:770–781. 33786357 10.1016/j.spc.2020.12.029 PMC7994925

[CR6] AlQhtani FM (2025) Knowledge management for research innovation in universities for sustainable development: a qualitative approach. Sustainability 17(6):2481.

[CR7] Amador-Jiménez M, Millner N (2021) Militarisation under COVID-19: understanding the differential impact of lockdown on the forests of Colombia. Front Hum Dyn 3:769365.

[CR8] Anthonia AN, Bello YA, Oliatan OM, Macaulay IA (2021) Reviewing the links between climate change and resource conflict. Glob J Pure Appl Sci 27(2):152–243.

[CR9] Anuradha M, Rao KR (2023) Overview on monkey pox an emerging viral infection. A review of literature. Eur J Mol Clin Med 10(1):2023

[CR10] Atin V, Lintangah W (2023) Impact and mitigation measures of COVID-19 towards food security through participation in forest management by community in Sook, Keningau District, Sabah. Soc 7(1):26–42.

[CR11] Bai PJ, Wardle J, Steel A, Adams J (2019) Utilisation of and attitude towards traditional and complementary medicine among Ebola survivors in Sierra Leone. Medicina 55(7):387. 31323758 10.3390/medicina55070387 PMC6681324

[CR12] Baldé AM, Traoré MS, Baldé MA, Barry MS, Diallo A, Camara M, Traoré S, Kouyaté M, Ouo-Ouo S, Myanthé AL (2016) Ethnomedical and ethnobotanical investigations on the response capacities of Guinean traditional health practioners in the management of outbreaks of infectious diseases: the case of the Ebola virus epidemic. J Ethnopharmacol 182:137–149. 26900129 10.1016/j.jep.2016.02.021

[CR13] Barbiero VK (2020) Ebola: a hyperinflated emergency. Glob Health Sci Pr 8(2):178–182. 10.9745/GHSP-D-19-00422 PMC7326525 32430358

[CR14] Behera AK, Kumar PR, Priya MM, Ramesh T, Kalle R (2022) The impacts of COVID-19 lockdown on wildlife in Deccan Plateau, India. Sci Total Environ 822:153268. 35074387 10.1016/j.scitotenv.2022.153268 PMC8782731

[CR15] Bijaya GC, Cheng S, Xu Z, Bhandari J, Wang L, Liu X (2016) Community forestry and livelihood in Nepal: a review. J Anim Plant Sci 26(1): 1−12

[CR16] Brancalion PHS, Broadbent EN, De-Miguel S, Cardil A, Rosa MR, Almeida CT, Almeida DRA, Chakravarty S, Zhou M, Gamarra JGP (2020) Emerging threats linking tropical deforestation and the COVID-19 pandemic. Perspect Ecol Conserv 18(4):243–246. 33020748 10.1016/j.pecon.2020.09.006 PMC7526655

[CR17] Brousseau E, Garrouste P, Raynaud E (2011) Institutional changes: alternative theories and consequences for institutional design. J Econ Behav Organ 79(1–2):3–19.

[CR18] Chang MS, Ho BC, Chan KL (1991) Efficacy of diethylcarbamazine and pirimiphos-methyl residual spraying in controlling brugian filariasis. Trop Med Parasitol Organ Dtsch Tropenmedizinische Ges Dtsch Ges Fur Tech Zusammenarbeit (GTZ) 42(2):95–102. 1680246

[CR19] Chao S (2012) Forest peoples: numbers across the world. Forest Peoples Programme Moreton-in-Marsh, UK

[CR20] Chirwa PW, Adeyemi O (2019) Deforestation in Africa: Implications on Food and Nutritional Security. In: Leal Filho W, Azul A, Brandli L, Özuyar P, Wall T (eds) Zero Hunger. Encyclopedia of the UN Sustainable Development Goals. Springer, Cham. 10.1007/978-3-319-69626-3_62-1

[CR21] Dakubo CY (2010) Ecosystems and human health: a critical approach to ecohealth research and practice. Springer Science & Business Media

[CR22] Das BK (2022) How to make forest governance reform real? Formal and informal institutions during implementation of the Forest Rights Act 2006 in India. Environ Dev 43:100729.

[CR23] Davis EC, Ivanic M, Sohngen B (2025) Avoiding global deforestation by taxing land in agricultural production: the implications for global markets. Carbon Balance Manag 20(1):5. 40082343 10.1186/s13021-025-00291-7 PMC11907821

[CR24] De Meyer E, Van Damme P, de la Peña E, Ceuterick M (2022) A disease like any other’traditional, complementary and alternative medicine use and perspectives in the context of COVID-19 among the Congolese community in Belgium. J Ethnobiol Ethnomed 18(1):29. 35392948 10.1186/s13002-022-00530-y PMC8988475

[CR25] Derks J, Giessen L, Winkel G (2020) COVID-19-induced visitor boom reveals the importance of forests as critical infrastructure. Policy Econ 118(July):102253. 10.1016/j.forpol.2020.102253. 10.1016/j.forpol.2020.102253 PMC7355319 32834768

[CR26] Devaux CA, Mediannikov O, Medkour H, Raoult D (2019) Infectious disease risk across the growing human-non human primate interface: a review of the evidence. Front Public Health 7:472360. 10.3389/fpubh.2019.00305 PMC6849485 31828053

[CR27] Emily F, Unruh J (2008) Demarcating forest, containing disease: Land and HIV/AIDS in southern Zambia. Popul Environ 29(3–5):108–132. 10.1007/s11111-008-0067-8.

[CR28] FAO (2020) The State of the world’s Forests: forest pathways to sustainable development. 10.1007/978-981-19-5478-8_1

[CR29] FAO and UNEP (2020) The State of the World’s Forests 2020. forests, biodiversity and people. Rome. Retrieved from: http://www.fao.org/3/ca8642en/CA8642EN.pdf. Accessed 4 Jun 2022

[CR30] Fleetwood S (2008) Institutions and social structures 1. J Theory Soc Behav 38(3):241–265.

[CR31] Garcia CA, Savilaakso S, Verburg RW, Gutierrez V, Wilson SJ, Krug CB, Sassen M, Robinson BE, Moersberger H, Naimi B (2020) The global forest transition as a human affair. One Earth 2(5):417–428.

[CR32] Genschel P (1997) The dynamics of inertia: institutional persistence and change in telecommunications and health care. Governance 10(1):43–66.

[CR33] Gentle P, Maraseni TN, Paudel D, Dahal GR, Kanel T, Pathak B (2020) Effectiveness of community forest user groups (CFUGs) in responding to the 2015 earthquakes and COVID-19 in Nepal. Res Glob 2:100025.

[CR34] Gerschewski J (2021a) Explanations of institutional change: reflecting on a “missing diagonal. Am Polit Sci Rev 115(1):218–233.

[CR35] Gerschewski J (2021b) Explanations of institutional change: reflecting on a missing diagonal. Am Polit Sci Rev 115(1):218–233. 10.1017/S0003055420000751.

[CR36] Giessen L, Buttoud G (2014) Assessing forest governance-analytical concepts and their application. Policy Econ 49:1–71.

[CR37] Giessen L, Krott M, Möllmann T (2014) Increasing representation of states by utilitarian as compared to environmental bureaucracies in international forest and forest–environmental policy negotiations. Policy Econ 38:97–104.

[CR38] Goguen A, Bolten C (2017) Ebola through a glass, darkly: ways of knowing the state and each other. Anthropol Q 90(2):423–449

[CR39] Gossé JK, Heighton SP, Gaubert P, Bi SG (2023) Long-term effect of the COVID-19 lockdown on the dynamics of the bushmeat trade in West Africa (Côte d’Ivoire). Preprint at 10.22541/au.167286378.87448559/v1.

[CR40] Greif A, Laitin DD (2004) A theory of endogenous institutional change. Am Polit Sci Rev 98(4):633–652.

[CR41] Gully PR (2020) Pandemics, regional outbreaks, and sudden-onset disasters. Health Manag Forum 33(4):164–169. 10.1177/0840470420901532. 10.1177/0840470420901532 PMC7201195 32022584

[CR42] Gusenbauer M (2020) Which Academic Search Systems Are Suitable for Systematic Reviewsor Meta-Analyses ? Evaluating Retrieval Qualities of Google Scholar , PubMed, and 26 Other Resources. pp. 181–217. 10.1002/jrsm.1378 PMC7079055 31614060

[CR43] Harrison S, Kivuti-Bitok L, Macmillan A, Priest P (2019) EcoHealth and One Health: a theory-focused review in response to calls for convergence. Environ Int 132(August):105058. 10.1016/j.envint.2019.105058. 31473414 10.1016/j.envint.2019.105058

[CR44] Hayman DTS, Bowen RA, Cryan PM, McCracken GF, O’shea TJ, Peel AJ, Gilbert A, Webb CT, Wood JLN (2013) Ecology of zoonotic infectious diseases in bats: current knowledge and future directions. Zoonoses Public Health 60(1):2–21. 22958281 10.1111/zph.12000 PMC3600532

[CR45] Hodgson GM (2006) What are institutions?. J Econ Issues 40(1):1–25.

[CR46] Hossain MT, Lima TR, Ela MZ, Khan L, Ahmed F, Al Masud A, Rahman K-S, Jahan N, Rahman SM, Islam MN (2022) Livelihood challenges and healthcare-seeking behavior of fishermen amidst the COVID-19 pandemic in the Sundarbans mangrove forest of Bangladesh. Aquaculture 546:737348. 34493879 10.1016/j.aquaculture.2021.737348 PMC8414286

[CR47] Houghton RA, Nassikas AA (2018) Negative emissions from stopping deforestation and forest degradation globally. Glob Change Biol 24(1):350–359. 10.1111/gcb.13876 28833909

[CR48] Hsieh H-F, Shannon SE (2005) Three approaches to qualitative content analysis. Qual Health Res 15(9):1277–1288. 16204405 10.1177/1049732305276687

[CR49] ILO (2020) Impact of COVID-19 on the forest sector. International Labour Organisation (ILO) Sectoral Brief. May 2019, p 6–10

[CR50] Karesh WB, Dobson A, Lloyd-Smith JO, Lubroth J, Dixon MA, Bennett M, Aldrich S, Harrington T, Formenty P, Loh EH (2012) Ecology of zoonoses: natural and unnatural histories. Lancet 380(9857):1936–1945. 23200502 10.1016/S0140-6736(12)61678-X PMC7138068

[CR51] Keele BF, Van Heuverswyn F, Li Y, Bailes E, Takehisa J, Santiago ML, Bibollet-Ruche F, Chen Y, Wain LV, Liegeois F (2006) Chimpanzee reservoirs of pandemic and nonpandemic HIV-1. Science 313(5786):523–526. 16728595 10.1126/science.1126531 PMC2442710

[CR52] Khadka D, Dhamala MK, Li F, Aryal PC, Magar PR, Bhatta S, Thakur MS, Basnet A, Cui D, Shi S (2021) The use of medicinal plants to prevent COVID-19 in Nepal. J Ethnobiol Ethnomed 17(1):1–17. 10.1186/s13002-021-00449-w. 33832492 10.1186/s13002-021-00449-w PMC8027983

[CR53] Kimengsi JN, Balgah RA (2021) Colonial hangover and institutional bricolage processes in forest use practices in Cameroon. Policy Econ 125:102406.

[CR54] Kimengsi JN, Bhusal P (2022) Community forestry governance: lessons for Cameroon and Nepal. Soc Nat Resour 35(4):447–464.

[CR55] Kimengsi JN, Mukong AK (2023) Forest resource endogenous cultural institutions in rural Cameroon: compliance determinants and policy implications. J Environ Plan Manag 66(7):1579–1600.

[CR56] Kimengsi JN, Mukong AK, Balgah RA (2020) Livelihood Diversification and Household Well-Being: Insights and Policy Implications for Forest-Based Communities in Cameroon. Soc Nat Resour 33(7):876–895. 10.1080/08941920.2020.1769243.

[CR57] Kimengsi JN, Mukong AK, Balgah RA (2023) Actors and institutional change determinants in the santchou landscape of Cameroon. Environ Dev 45:100778.

[CR58] Kimengsi JN, Mukong AK, Forje GW, Giessen L (2024) Institutional change pathways and implications for forest resource use in the Bakossi landscape of Cameroon. J Nat Conserv 78:126567.

[CR59] Kimengsi JN, Mukong AK, Giessen L, Pretzsch J (2022) Institutional dynamics and forest use practices in the Santchou Landscape of Cameroon. Environ Sci Policy 128:68–80.

[CR60] Kimengsi JN, Owusu R, Charmakar S, Manu G, Giessen L (2023) A global systematic review of forest management institutions: towards a new research agenda. Landsc Ecol 38(2):307–326. 10.1007/s10980-022-01577-8. 36589773 10.1007/s10980-022-01577-8 PMC9789374

[CR61] Kimengsi JN, Owusu R, Djenontin INS, Pretzsch J, Giessen L, Buchenrieder G, Pouliot M, Acosta AN (2022) What do we (not) know on forest management institutions in sub-Saharan Africa? A regional comparative review. Land Use Policy 114:105931.

[CR62] Koju NP, Kandel RC, Acharya HB, Dhakal BK, Bhuju DR (2021) COVID-19 lockdown frees wildlife to roam but increases poaching threats in Nepal. Ecol Evol 11(14):9198–9205. 34306616 10.1002/ece3.7778 PMC8293707

[CR63] Koning EA (2016) The three institutionalisms and institutional dynamics: Understanding endogenous and exogenous change. J Public Policy 36(4):639–664. 10.1017/S0143814X15000240.

[CR64] Krott M, Bader A, Schusser C, Devkota R, Maryudi A, Giessen L, Aurenhammer H (2014) Actor-centred power: the driving force in decentralised community based forest governance. Policy Econ 49:34–42. 10.1016/j.forpol.2013.04.012.

[CR65] Kumar V, Pruthvishree B, Pande T, Sinha D, Singh B, Dhama K, Malik YS (2020) SARS-CoV-2 (COVID-19): zoonotic origin and susceptibility of domestic and wild animals. J Pure Appl Microbiol 14(suppl 1):741–747.

[CR66] Kuuwill A, & Kimengsi J N (2024) The COVID-19 pandemic and dynamics of livelihood assets in the Kwahu South District of Ghana: determinants and policy implications. Development in Practice, 34(5), 611-632.

[CR67] Kuuwill A, Kimengsi JN (2023) AQ16 COVID-19 pandemic and livelihood assets dynamics in the Kwahu South District of Ghana: determinants and policy implications

[CR68] Kuuwill A, Kimengsi JN, Campion, BB (2022) Pandemic-induced shocks and shifts in forest-based livelihood strategies: learning from COVID-19 in the Bia West District of Ghana. Environ Res Lett 17(6):064033.

[CR69] Kuuwill A, Kimengsi JN, & Giessen, L (2025) Compliance with forest management institutions in Ghana: Communities’ perceptions and actor constellations under pandemic and epidemic health shocks. Trees, Forests and People, 100970.

[CR70] Kuuwill A, Kimengsi JN, Natcher D, Agyepong L, Acquaah G, Ampomah S, Dasmani I, Darfor KN, Ofori PE (2024) Health shocks and rural farmers credit access shifts in Sub-Saharan Africa: evidence from the Kwahu Afram Plains South District, Ghana. Environ Chall 15:100924

[CR71] Lainson R (1983) The American leishmaniases: some observations on their ecology and epidemiology. Trans R Soc Trop Med Hyg 77(5):569–596. 6197791 10.1016/0035-9203(83)90185-2

[CR72] Larasatie P, Fitriastuti T, Yovi EY, Purnomo H, Nurrochmat DR (2022) COVID-19 anxiety as a moderator of the relationship between organizational change and perception of organizational politics in forestry public sector. Forests 13(2):356.

[CR73] Laudari HK, Pariyar S, Maraseni T (2021) COVID-19 lockdown and the forestry sector: Insight from Gandaki province of Nepal. Policy Econ 131:102556. 10.1016/j.forpol.2021.102556 PMC8419634 34512124

[CR74] Lebel J (2003) Health: an ecosystem approach; the issue, case studies, lessons and recommendations. IDRC, Ottawa, ON, CA

[CR75] Li K, Rollins J, Yan E (2018) Web of Science use in published research and review papers 1997–2017: a selective, dynamic, cross-domain, content-based analysis. Scientometrics 115(1):1–20. 29527070 10.1007/s11192-017-2622-5 PMC5838136

[CR76] Li S, Sparrow SN, Otto FEL, Rifai SW, Oliveras I, Krikken F, Anderson LO, Malhi Y, Wallom D (2021) Anthropogenic climate change contribution to wildfire-prone weather conditions in the Cerrado and Arc of deforestation. Environ Res Lett 16(9):94051.

[CR77] Lomazzi V (2023) The cultural roots of violence against women: individual and institutional gender norms in 12 countries. Soc Sci 12(3):117.

[CR78] Mahoney J, Thelen K (2010) A theory of gradual institutional change. In: Explaining institutional change: ambiguity, agency, power, vol 1. p 1

[CR79] Maraseni T, Poudyal BH, Aryal K, Laudari HK (2022) Impact of COVID-19 in the forestry sector: a case of lowland region of Nepal. Land Use Policy 120:106280. 35880191 10.1016/j.landusepol.2022.106280 PMC9300748

[CR80] Marrelli MT, Malafronte RS, Sallum MAM, Natal D (2007) Kerteszia subgenus of Anopheles associated with the Brazilian Atlantic rainforest: current knowledge and future challenges. Malar J 6:1–8. 17880709 10.1186/1475-2875-6-127 PMC2082038

[CR81] Mbiba M, Collinson M, Hunter L, Twine W (2018) Social capital is subordinate to natural capital in buffering rural livelihoods from negative shocks: insights from rural South Africa. J Rural Stud. 10.1016/j.jrurstud.2018.12.012

[CR82] Mcginlay J, Gkoumas V, Holtvoeth J, Fuertes A, Bazhenova E, Benzoni A, Botsch K, Martel CC, Carrillo C, Cervera I, Chaminade G, Doerstel J (2020) The impact of COVID-19 on the management of european protected areas and policy implications. Forests 1214(11):11.

[CR83] Mengist W, Soromessa T, Legese G (2020) Method for conducting systematic literature review and meta-analysis for environmental science research. MethodsX 7:100777. 31993339 10.1016/j.mex.2019.100777 PMC6974768

[CR84] Miller-Rushing AJ, Athearn N, Blackford T, Brigham C, Cohen L, Cole-Will R, Edgar T, Ellwood ER, Fisichelli N, Pritz CF (2021) COVID-19 pandemic impacts on conservation research, management, and public engagement in US national parks. Biol Conserv 257:109038. 34580547 10.1016/j.biocon.2021.109038 PMC8459301

[CR85] Morens DM, Taubenberger JK, Fauci AS (2009) The persistent legacy of the 1918 influenza virus. N Engl J Med 361(3):225–229. 19564629 10.1056/NEJMp0904819 PMC2749954

[CR86] Mufunda J, Ndambakuwa Y, Munodawafa D, Kobie A (2016) Is a total ban on business and consumption of bushmeat a sustainable end game for Ebola outbreak in West Africa: but why now. Public Health Open J 1(1):4–7.

[CR87] Munck af Rosenschöld J, Rozema JG, Frye-Levine LA (2014) Institutional inertia and climate change: a review of the new institutionalist literature. Wiley Interdiscip Rev Clim Change 5(5):639–648.

[CR88] Ndlovu M, Matipano G, Miliyasi R (2021) An analysis of the effect of COVID-19 pandemic on wildlife protection in protected areas of Zimbabwe in 2020. Sci Afr 14:e01031. 34746521 10.1016/j.sciaf.2021.e01031 PMC8557114

[CR89] Ndlovu T, Mjimba V (2021) Drought risk-reduction and gender dynamics in communal cattle farming in southern Zimbabwe. Int J Disaster Risk Reduct 58:102203.

[CR90] Newton P, Kinzer AT, Miller DC, Oldekop JA, Agrawal A (2020) The number and spatial distribution of forest-proximate people globally. One Earth 3(3):363–370. 10.1016/j.oneear.2020.08.016.

[CR91] Newton P, Miller DC, Byenkya MAA, Agrawal A (2016) Who are forest-dependent people? A taxo nomy to aid livelihood and land use decision-making in forested regions. Land Use Policy 57:388–395.

[CR92] Nysten-Haarala S (2013) Creating trust in institutions in Russian forest localities. Policy Econ 31:12–19.

[CR93] Onyekuru NA, Ume CO, Ezea CP, Chukwuma Ume NN (2020) Effects of Ebola virus disease outbreak on bush meat enterprise and environmental health risk behavior among households in South-east Nigeria. J Prim Prev 41:603–618. 33222018 10.1007/s10935-020-00619-8 PMC7680257

[CR94] Ostrom E (2005) Understanding institutional diversity. Princeton University Press, p. 375

[CR95] Owusu R, Kimengsi JN, Giessen L (2024) Institutional change and compliance in forest landscape restoration governance: insights from the Western Highlands of Cameroon. J Land Use Sci 19(1):36–58.

[CR96] Pachetti M, Marini B, Giudici F, Benedetti F, Angeletti S, Ciccozzi M, Masciovecchio C, Ippodrino R, Zella D (2020) Impact of lockdown on Covid-19 case fatality rate and viral mutations spread in 7 countries in Europe and North America. J Transl Med 18:1–7. 32878627 10.1186/s12967-020-02501-x PMC7463225

[CR97] Paumgarten F, Locatelli B, Witkowski ETF, Vogel C (2020) Prepare for the unanticipated: Portfolios of coping strategies of rural households facing diverse shocks. J Rural Stud 80:91–100

[CR98] Peters BG (2014) Implementation structures as institutions. Public Policy Adm 29(2):131–144.

[CR99] Pham TT, Tang TKH, Dang HP, Nguyen TKN, Hoang TL, Tran NMH, Nguyen TTA, Nguyen TVA, Valencia I (2022) Policymaker perceptions of COVID-19 impacts, opportunities and challenges for sustainable wildlife farm management in Vietnam. Environ Sci Policy 136:497–509. 35855780 10.1016/j.envsci.2022.07.017 PMC9279387

[CR100] Pichlerová M, Önkal D, Bartlett A, Výbošťok J, Pichler V (2021) Variability in forest visit numbers in different regions and population segments before and during the COVID-19 pandemic. Int J Environ Res Public Health 18(7):3469. 33810557 10.3390/ijerph18073469 PMC8037241

[CR101] Pretzsch J, Darr D, Uibrig H, Auch E, 2014. Forests and Rural Development. Springer-Verlage, Berlin Heidelberg. Shackleton S, Delang C. O, & Angelsen A. (2011). From subsistence to safety nets and cash income: exploring the diverse values of non-timber forest products for livelihoods and poverty alleviation. In Non-timber forest products in the global context Springer, Berlin, Heidelberg p 55-81.

[CR102] Prothero RM (1999) Malaria, forests and people in Southeast Asia. Singap J Trop Geogr 20(1):76–85.

[CR103] Rahimian M, Masoudi Rad M, Zareei H (2022) The effects of the Covid-19 pandemic on ecotourism, a study from West of Iran. Front Public Health 10:983025. 36148340 10.3389/fpubh.2022.983025 PMC9485483

[CR104] Rahman MT, Sobur MA, Islam MS, Ievy S, Hossain MJ, El Zowalaty ME, Rahman AMMT, Ashour HM (2020) Zoonotic diseases: etiology, impact, and control. Microorganisms 8(9):1405. 32932606 10.3390/microorganisms8091405 PMC7563794

[CR105] Rana P, Fleischman F (2023) Indian forest governance during the COVID-19 pandemic. Int Rev 25(1):105–120.

[CR106] Saikia M, Mahanta R (2025) An assessment of the nature and structure of institutions in the char areas of Assam, India. Asian Ethn 1–22.

[CR107] Sapkota LM, Silori CS, Dangal SP, Than MM, Sokchea T, Chhneang K, Thu AK, Van Chau T, Katwal N (2022) Beyond the biophysical: contribution of community forestry in building social-ecological resilience. In: Forest dynamics and conservation: science, innovations and policies. p 187–211

[CR108] Saxena A, Dutta A, Fischer HW, Saxena AK, Jantz P (2021) Forest livelihoods and a “green recovery” from the COVID-19 pandemic: insights and emerging research priorities from India. Policy Econ 131:102550. 10.1016/j.forpol.2021.102550 PMC9760494 36570104

[CR109] Schusser C, Krott M, Movuh MCY, Logmani J, Devkota RR, Maryudi A, Salla M, Bach ND (2015) Forest Policy and Economics Powerful stakeholders as drivers of community forestry—results of an international study. Policy Econ 58:92–101. 10.1016/j.forpol.2015.05.011.

[CR110] Sebego RJ, Atlhopheng JR, Chanda R, Mulale K, Mphinyane W (2019) Land use intensification and implications on land degradation in the Boteti area: Botswana. Afr Geogr Rev 38(1):32–47. 10.1080/19376812.2017.1284599.

[CR111] Shackleton S, Delang CO, Angelsen A (2011) From subsistence to safety nets and cash income: exploring the diverse values of non-timber forest products for livelihoods and poverty alleviation. In Non-timber forest products in the global context. Springer, p 55–81

[CR112] Sinclair S, Smetters KA (2004) Health shocks and the demand for annuities. Congressional Budget Office, Washington, DC

[CR113] Soliku O, Kyiire B, Mahama A, Kubio C (2021) Tourism amid COVID-19 pandemic: impacts and implications for building resilience in the ecotourism sector in Ghana’s Savannah region. Heliyon 7(9):1−10 10.1016/j.heliyon.2021.e07892 PMC8412235 34493990

[CR114] Tamakloe MD, Kuuwill A, Osumanu I, Siripi H (2025) Mathematical modelling and time series clustering of Mpox outbreak: A comparative study of the top 10 affected countries and implications for future outbreak management. Glob Epidemiol 100214. 10.1016/j.gloepi.2025.100214 PMC12332957 40787209

[CR115] Tiwari R, Dhama K, Sharun K, Iqbal, Yatoo M, Malik YS, Singh R, Michalak I, Sah R, Bonilla-Aldana DK, Rodriguez-Morales AJ (2020) COVID-19: animals, veterinary and zoonotic links. Vet Q 40(1):169–182. 32393111 10.1080/01652176.2020.1766725 PMC7755411

[CR116] Turner MD (1999) Conflict, environmental change, and social institutions in dryland Africa: limitations of the community resource management approach. Soc Nat Resour 12(7):643–657.

[CR117] Vale MM, Berenguer E, de Menezes MA, de Castro EBV, de Siqueira LP, Rita de Cássia QP (2021) The COVID-19 pandemic as an opportunity to weaken environmental protection in Brazil. Biol Conserv 255:108994. 33568834 10.1016/j.biocon.2021.108994 PMC7862926

[CR118] van Kooten GC, Schmitz A (2022) COVID-19 impacts on US lumber markets. Policy Econ 135:102665. 10.1016/j.forpol.2021.102665 PMC8646330 34899041

[CR119] Wakjira DT, Fischer A, Pinard MA (2013) Governance change and institutional adaptation: a case study from Harenna Forest, Ethiopia. Environ Manag 51:912–925. 10.1007/s00267-013-0017-9 23354873

[CR120] WHO (2024) WHO COVID-19 dashboard. Sci Rep. https://data.who.int/dashboards/covid19/deaths?n=c. Accessed 22 Mar 2024

[CR121] Xiao X, Newman C, Buesching CD, Macdonald DW, Zhou Z-M (2021) Animal sales from Wuhan wet markets immediately prior to the COVID-19 pandemic. Sci Rep. 11(1):1–7. 34099828 10.1038/s41598-021-91470-2 PMC8184983

[CR122] Yeboah-Assiamah E, Muller K, Domfeh KA (2019) Two sides of the same coin: formal and informal institutional synergy in a case study of wildlife governance in Ghana. Soc Nat Resour 32(12):1364–1382.

