# Supplementary material for: Change in Formal and Informal Forest Management Institutions Induced by Health Shocks—A Global Systematic Review
Source: Environ Manage. 2025 Sep 3;75(12):3565–85. doi: 10.1007/s00267-025-02250-x (PMC12575515; doi:10.1007/s00267-025-02250-x)
Supplement: Supplementary file 1 — Appendix [file 267_2025_2250_MOESM1_ESM.docx]

Appendix

Supplementary Table 1: Search terms used to develop search strings for database search

| **Institutions** | **Institutions** | **Change** | **Forest** | **Management** | **Health shock** |
| --- | --- | --- | --- | --- | --- |
| Law  Statute  Regulation  Rule  Ordinance  Legislation  Code  Act  Statutory law  Legal code  Principle  Mandate  Dictate  Decree  Order  Edict  Bylaw  Canon | Custom  Beliefs  Myths  Superstition  Culture  Tradition  Norm  Folkway  Unwritten rule  Practice  Habit  Ritual  Mores  Ethos  Attitude  Code of conduct  Behavioural norm  Social expectations  Accepted behavior | Alteration  Modification  Variation  Shift  Transformation  Adaptation  Adjustment  Metamorphosis  Evolution  Transition  Divergence  Fluctuation  Amendment  Revision  Development | Deciduous forest  Forest  Biodiversity  ecosystem  Ecosystem  Vegetation  Vegetation cover  Rainforest  Timber  NTFP  Wildlife  Game  Medicinal plants | Governance  Control  Access  Administration  Supervision  Stewardship  Operation | Disease outbreak  Spillover  Health crises  Health setbacks  Public health emergence  Health disruption  Health disaster  Health disturbance |

Supplementary Table 2: regional and subregional distribution of number of articles and cases

| Continent | Number of articles | Cases | Cases classification |
| --- | --- | --- | --- |
| **Africa** |  |  | **West Africa (19)** |
| Cameroon | 2 | 4 | Ghana 5 |
| Central Africa Republic | 1 | 2 | Guinea 1 |
| DRC Congo | 1 | 1 | Ivory Coast 5 |
| Gabon | 1 | 1 | Nigeria 2 |
| Ghana | 3 | 5 | Sierra Leon 6 |
| Guinea | 1 | 1 | **East Africa (8)** |
| Ivory Coast | 1 | 5 | Malawi 1 |
| Malawi | 1 | 1 | Uganda 7 |
| Namibia | 1 | 5 | **Central Africa (8)** |
| Nigeria | 2 | 2 | Cameroon 4 |
| Sierra Leone | 5 | 6 | Central Africa Rep 2 |
| Uganda | 6 | 7 | DRC 1 |
| Zimbabwe | 3 | 4 | Gabon 1 |
| **Total** | **28** | **44** | **Southern Africa (9)** |
|  |  |  | Namibia 5 |
|  |  |  | Zimbabwe 4 |
| **Asia** |  |  |  |
| Bangladesh | 1 | 1 | **South Asia (14)** |
| Cambodia |  | 2^ab^ | Bangladesh 1 |
| Myanmar |  | 1^b^ | India 4 |
| Vietnam | 2 | 6^ab^ | Nepal 9 |
| Nepal | 6 | 9^b^ | **Southeast Asia (19)** |
| China | 1 | 1 | Cambodia 2 |
| India | 3 | 4 | Indonesia 6 |
| Indonesia | 6 | 6 | Myanmar 1 |
| Iran | 2 | 2 | Philippines 1 |
| Japan | 1 | 1 | Thailand 1 |
| Malaysia | 1 | 1 | Vietnam 6 |
| Philippine | 1 | 1 | Laos 1 |
| Republic of Korea | 1 | 1 | Malaysia 1 |
| Thailand | 1 | 1 | **East Asia (3)** |
| Turkey | 1 | 1 | China1 |
| Laos |  | 1^a^ | Japan 1 |
| Cross country paper^a^ | 1 |  | Republic of Korea 1 |
| Cross country paper^b^ | 1 |  | **Western Asia (3)** |
| **Total** | **29** | **39** | Iran 2 |
|  |  |  | Turkey 1 |
| **Europe** |  |  | **Western Europe (4)** |
| Belgium | 1 | 1 | Belgium 1 |
| Czech Republic | 1 | 1 | Germany 1 |
| Slovak | 1 | 1 | Spain 1 |
| United Kingdom^c^ |  | 1 | Italy 1 |
| Germany^c^ |  | 1 | **Central Europe (3)** |
| Spain^c^ |  | 1 | Czech Republic 1 |
| Italy^c^ |  | 1 | Slovakia 1 |
| Estonia^c^ |  | 1 | Poland 1 |
| Poland^c^ |  | 1 | **Northern Europe (2)** |
| Slovnia^c^ |  | 1 | United Kingdom1 |
| Sweden^c^ |  | 1 | Sweden 1 |
| Cross country paper^c^ | 1 |  | **Eastern Europe (2)** |
|  |  |  | Estonia 1 |
|  |  |  | Slovenia 1 |
| **North America** |  |  | **North America (6)** |
| Canada | 1 | 1 | Canada 1 |
| USA | 2 | 2 | USA 2 |
| Gaugamela^d^ |  | 1 | Mexico 2 |
| Mexico^d^ | 1 | 2 | (cta) Gautamela 1 |
| **EUR+NA** | **8** | **17** |  |
| **Latin America** |  |  | **Andean Region (5)** |
| Brazil | 1 | 1 | Colombia (3) |
| Colombia | 2 | 3 | Equador (1) |
| Equador^d^ |  | 1 | Peru (1) |
| Argentina^d^ |  | 1 | **Southern Cone (2)** |
| Peru | 1 | 1 | Argentina (1) |
| Cross country paper^d^ | 1 |  | Brazil (1) |
| **Total** | **5** | **7** |  |
